# The EICAT+ framework enables classification of positive impacts of alien taxa on native biodiversity

**DOI:** 10.1371/journal.pbio.3001729

**Published:** 2022-08-16

**Authors:** Giovanni Vimercati, Anna F. Probert, Lara Volery, Ruben Bernardo-Madrid, Sandro Bertolino, Vanessa Céspedes, Franz Essl, Thomas Evans, Belinda Gallardo, Laure Gallien, Pablo González-Moreno, Marie Charlotte Grange, Cang Hui, Jonathan M. Jeschke, Stelios Katsanevakis, Ingolf Kühn, Sabrina Kumschick, Jan Pergl, Petr Pyšek, Loren Rieseberg, Tamara B. Robinson, Wolf-Christian Saul, Cascade J. B. Sorte, Montserrat Vilà, John R. U. Wilson, Sven Bacher

**Affiliations:** 1 Department of Biology, University of Fribourg, Fribourg, Switzerland; 2 Department of Integrated Biology, Estación Biológica de Doñana (EBD), CSIC, Sevilla, Spain; 3 Department of Life Sciences and Systems Biology, University of Turin, Torino, Italy; 4 Laboratory of Aquatic Ecology, Estación Biológica de Doñana (EBD), CSIC, Sevilla, Spain; 5 Bioinvasions, Global Change, Macroecology-Group, Department of Botany and Biodiversity Research, University of Vienna, Vienna, Austria; 6 Ecologie Systématique et Evolution, Université Paris-Saclay, Gif-sur-Yvette, France; 7 Institute of Biology, Freie Universität Berlin, Berlin, Germany; 8 Leibniz Institute of Freshwater Ecology and Inland Fisheries (IGB), Berlin, Germany; 9 Berlin-Brandenburg Institute of Advanced Biodiversity Research (BBIB), Berlin, Germany; 10 Instituto Pirenaico de Ecología (IPE), CSIC, Zaragoza, Spain; 11 Univ. Grenoble Alpes, Univ. Savoie Mont Blanc, CNRS, LECA, Grenoble, France; 12 Department of Forest Engineering, University of Córdoba, Córdoba, Spain; 13 Centre for Invasion Biology, Department of Mathematical Sciences, Stellenbosch University, Stellenbosch, South Africa; 14 Biodiversity Informatics Unit, African Institute for Mathematical Sciences, Cape Town, South Africa; 15 Department of Marine Sciences, University of the Aegean, Mytilene, Greece; 16 Department Community Ecology, Helmholtz Centre for Environmental Research—UFZ, Halle, Germany; 17 Department of Geobotany and Botanical Garden, Martin Luther University Halle-Wittenberg, Halle, Germany; 18 German Centre for Integrative Biodiversity Research (iDiv) Halle-Jena-Leipzig, Leipzig, Germany; 19 Centre for Invasion Biology, Department of Botany and Zoology, Stellenbosch University, Stellenbosch, South Africa; 20 Kirstenbosch Research Centre, South African National Biodiversity Institute, Cape Town, South Africa; 21 Department of Invasion Ecology, Institute of Botany, Czech Academy of Sciences, Průhonice, Czech Republic; 22 Department of Ecology, Faculty of Science, Charles University, Prague, Czech Republic; 23 Department of Botany and Biodiversity Research Centre, University of British Columbia, Vancouver, Canada; 24 Department of Ecology and Evolutionary Biology, University of California, Irvine, California, United States of America; 25 Department of Plant Biology and Ecology, University of Sevilla, Sevilla, Spain

## Abstract

Species introduced through human-related activities beyond their native range, termed alien species, have various impacts worldwide. The IUCN Environmental Impact Classification for Alien Taxa (EICAT) is a global standard to assess negative impacts of alien species on native biodiversity. Alien species can also positively affect biodiversity (for instance, through food and habitat provisioning or dispersal facilitation) but there is currently no standardized and evidence-based system to classify positive impacts. We fill this gap by proposing EICAT+, which uses 5 semiquantitative scenarios to categorize the magnitude of positive impacts, and describes underlying mechanisms. EICAT+ can be applied to all alien taxa at different spatial and organizational scales. The application of EICAT+ expands our understanding of the consequences of biological invasions and can inform conservation decisions.

## Abstract 2

Various schemes assess negative impacts of alien species on native biodiversity, but alien species can also positively affect biodiversity. This Consensus View proposes EICAT+, a scheme which uses five semi-quantitative scenarios to categorise positive impacts, describes underlying mechanisms, and can be applied to all alien taxa and across various invasion contexts.

## Introduction

Taxa introduced beyond the limits of their native range (termed alien taxa) are a primary driver of global environmental change [1,2]. Once introduced to new areas, alien taxa can cause changes to their recipient ecosystems [3,4] (Box 1, Supporting information A in S1 File). These changes, called environmental impacts [3], are generally estimated by quantifying how certain attributes (i.e., measurable features or components; Box 1) of the recipient ecosystem vary due to the presence and abundance of alien taxa [4]. Increases or decreases in specific ecosystem attributes (e.g., in population size of a native species) can thus be described from a value-free perspective as positive or negative impacts, respectively (Box 1). In addition, the same alien taxon can have negative impacts on certain attributes of native biodiversity and positive impacts on others, with different levels of magnitude and underlying mechanisms [3,5].

**Box 1. Glossary of impact-related terms.**

**Biodiversity attribute:** any feature or component of biological diversity that can be measured, quantified, and compared, such as the taxonomic richness, phylogenetic diversity, and functional diversity of a community or the abundance, total biomass, and average body size of individuals in a population. In EICAT and EICAT+, the biodiversity attributes to quantify impacts caused by alien taxa on native taxa are performance of individuals, population size, and area of occupancy [10,24,25,117].

- **Individual performance:** any measurable trait that affects the capacity of an individual organism to survive, gather resources, grow, or reproduce. Examples include body mass or size, number of offspring or seeds, physiological rates (such as rates of growth, respiration, calcification, etc.), and immunocompetence [23]. Note that changes in performance do not necessarily lead to changes in population size.
- **Population size:** the number of mature individuals—i.e., individuals known, estimated or inferred to be capable of reproduction [23]—in a population. Note that changes in population size do not necessarily lead to changes in area of occupancy.
- **Area of occupancy:** the area occupied by a certain taxon within its native range, excluding cases of vagrancy [58,59]. Area of occupancy can decrease as a consequence of local extinctions (negative impact on area of occupancy) and increase as a consequence of local reestablishment (positive impact on area of occupancy).

**Impact:** any change caused by an alien taxon to the recipient system. Impacts are generally quantified by measuring how certain attributes of the recipient system (such as the amount of nitrogen or the number of fish in a lake) change due to the introduction of the alien taxon. In EICAT+, only impacts on the individual performance, population size, and area of occupancy of a native species are considered.

- **Beneficial/harmful impact:** any change caused by an alien taxon to the recipient system that is perceived by humans as favorable/unfavorable for an entity of interest (e.g., a native species, a sentient individual, or a protected ecosystem; see also Supporting information A in S1 File) in accordance with specific values/interests/motivations [9] (Supporting information A and B in S1 File). The words “beneficial” and “harmful” are thus used here regardless of whether the impact involves an increase (“positive impact”; see definition below) or a decrease (“negative impact”; see definition below) in the measured attribute. In this sense, a positive impact on a certain biodiversity attribute is not necessarily valued as beneficial, and a negative impact is not necessarily valued as harmful. Note that harmful impacts are also termed as deleterious impacts by some authors [8,10].
- **Positive/negative impact:** any increasing/decreasing change caused by an alien taxon to one of the selected biodiversity attributes of the recipient system [3,4,8,9,60]. As “increasing change” we herein consider relative changes. So, for example, if the size of a native population would have decreased, or decreased to a greater extent, if an alien species had not been introduced (see also Supporting information C in S1 File for more details), then that alien species has caused a (relative) increase in native biodiversity. Unlike beneficial/harmful impacts, positive/negative impacts describe quantitative and directional variations that can be objectively and consistently measured, regardless of ethical values (value-free classification of impact [8]).

**Impact mechanism:** the way in which alien taxa cause changes in attributes of the recipient system. Examples of mechanisms include “predation” and “parasitism” in EICAT and “provisions of trophic resources” and “dispersal facilitation” in EICAT+ (see below and Supporting information D and E in S1 File).

Considerable effort has been devoted to study the direction, magnitude, and mechanisms underlying impacts of alien taxa on ecosystems [3,6]. This effort has been driven primarily by the urgent need to limit the harm that certain alien taxa cause to various entities of interest within recipient ecosystems, such as native species or habitats [7,8] (Supporting information A and B in S1 File). Various cultural value systems and motivations, however, determine which entities we are interested in, whether negative or positive changes in biodiversity attributes are perceived as harmful or beneficial [4,8,9] (Box 1, Supporting information B in S1 File), and, consequently, how alien taxa should be managed (Supporting information B in S1 File). Some people might want to primarily protect taxa in their native range [10,11], rescue range-contracting taxa through translocations [12], uphold ecosystem functions [13] or improve biodiversity indicators regardless of species origin [14]. Others will evaluate conservation actions in terms of the suffering caused, e.g., to sentient taxa [15], rather than in utilitarian terms [16]. Alien taxa can therefore be opposed, tolerated, or even promoted as a function of specific environmental and sociocultural contexts (Supporting information B in S1 File). Such a variety of approaches in conservation is not surprising, given that conservation targets are usually defined in accordance with different values and motivations [17–19]. Once these values and motivations are openly recognized, conservation-related disciplines such as invasion science can adopt standardized and systematic approaches to measure the degree to which specific entities of interest are affected by certain drivers of change (e.g., alien taxa), so that informed and coherent management decisions (e.g., prioritization) can be made. However, the majority of impact assessment frameworks developed and applied in invasion science do not consider the ways of assessing increases to native biodiversity [8], and the field has been criticized for focusing on, and overemphasizing, the harmful impacts of alien taxa [20–22].

**Fig 1:**
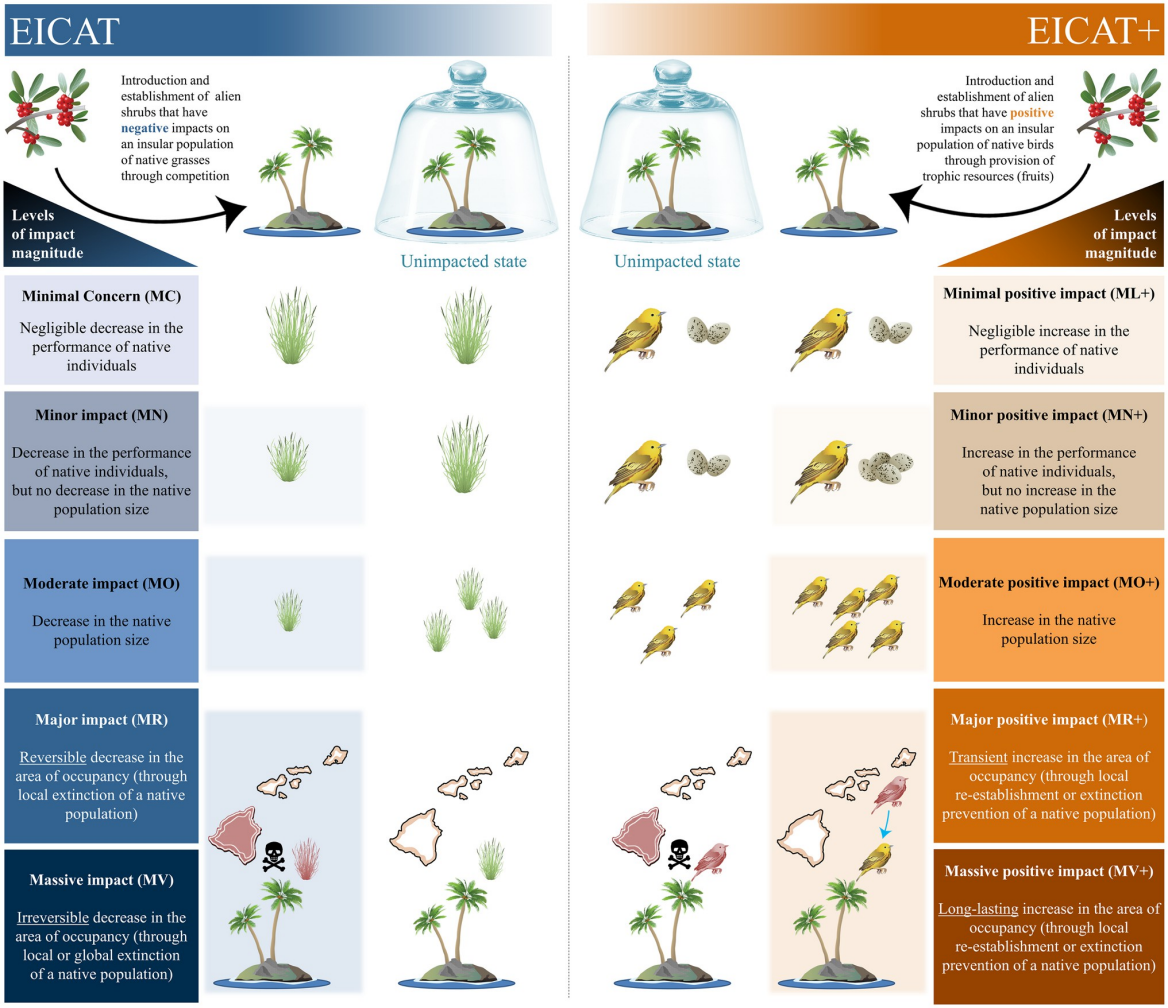
Illustration of EICAT and EICAT+ scenarios. Conceptual scheme for the 5 semiquantitative scenarios used in EICAT and EICAT+ to assess negative impacts (on the left) and positive impacts (on the right) caused by a focal alien taxon (here, a shrub species) on native taxa of interest (a grass species and a bird species). Black arrows indicate the introduction and establishment of the alien taxon into a recipient ecosystem. The blue arrow indicates the reestablishment or extinction prevention of a native taxon due to an alien taxon (see also Fig 2). Shaded red figures indicate locations unoccupied by the native taxon (e.g., because of local extinctions in EICAT). Symbols were courtesy of the Integration and Application Network, University of Maryland Center for Environmental Science (ian.umces.edu/media-library).

The International Union for Conservation of Nature (IUCN) Environmental Impact Classification for Alien Taxa (EICAT) framework was developed to categorize and assess negative impacts caused by alien taxa to native biodiversity at different levels of organization [10,23–26] (i.e., decreases in performance of individuals, population size, and area of occupancy through local population extinction). EICAT relies on the premise that the biogeographic origin of species matters in conservation. A clear distinction between native and alien taxa is justified by multiple conceptual and practical arguments associated with, for example, coevolutionary histories, ecological functions, or human responsibilities for nature [11,27,28]. Accordingly, EICAT entities of interest are native taxa, whose attributes recorded prior to the introduction or in the absence of an alien taxon are used as a baseline to assess impacts (Fig 1). Changes caused by alien taxa to abiotic ecosystem attributes, such as soil nitrogen content or water chemistry, are considered under the EICAT framework only if such changes lead to a decrease in attributes of native biodiversity. EICAT has been used to compare impact magnitudes of alien taxa across geographic regions and taxonomic groups [26,29–30] and to support evidence-based prioritization and other management decisions [31–33]. EICAT is conceptually and structurally related to the IUCN Red List of Threatened Species, with the Red List categorizing a focal native species based on its risk of extinction, and EICAT categorizing a focal alien taxon based on the degree to which it has negatively impacted native taxa [34].

Identifying which (and how) alien taxa decrease certain native biodiversity attributes does not fully capture the multifaceted environmental changes induced by the introduction of alien species; in particular, such attributes can also increase. For instance, an alien taxon may increase the performance of individuals or the size of a population of a native taxon by providing additional trophic resources or creating novel habitats that are suitable for the native taxon [5,35–37] (Fig 1). Clear and transparent evaluation of all impacts, negative and positive, on native taxa will help to achieve a better understanding of the dynamics and mechanisms of impacts. Such increased understanding would benefit scientists, managers, policymakers, and the general public; for example, it may enable the identification of societal or nature conservation conflicts associated with alien taxa and provide an objective evidence base to underpin discussions and debates [16,38]. The identification of conflicts between different conservation targets is particularly important when alien taxa harm certain native taxa while benefiting others, enabling robust and appropriate management interventions to reach conservation goals. We see 3 main reasons for developing a framework compatible with EICAT that assesses positive environmental impacts [8]:

1) Improving our understanding of interactions between alien and native taxa

The assessment of positive impacts of alien taxa on native biodiversity attributes can improve the understanding of transient and stable interactions between native and alien taxa [39]. Better understanding of multidirectional changes induced by alien taxa will shed light on how such interactions affect ecological and ecosystem dynamics [40], and provide valuable insights for adaptive management. For instance, the use of a common scheme to compare the outcomes of novel mutualistic interactions between alien and native taxa—e.g., the interaction between an alien plant and native arbuscular fungi [41], or between alien plants and native pollinators [42]—can help to identify which native taxa facilitate the establishment, or hamper the removal, of certain alien taxa. Similarly, a systematic assessment of the antagonistic interactions between alien taxa and their native consumers can help to identify which native species promote biotic resistance against established alien taxa [43,44] and might also be used in biological control [45,46].

2) Informing predictions of indirect, and potentially adverse, effects of alien taxa management

Improving the understanding of the positive impacts that an alien taxon causes on native biodiversity attributes can help to identify possible undesired effects that its removal might have on native communities [47]. For instance, the removal of aliens may release herbivorous pests or meso-predators controlled by alien predators [48], decrease the density of predators feeding on alien preys [49], or reduce the availability of predator-free refugia provided by an alien plant and exploited by endangered species [50].

3) Improving our understanding of the interactions between alien taxa and other drivers of global change

The assessment of positive environmental impacts can clarify the degree to which already established alien populations mitigate adverse effects of other global anthropogenic stressors, such as habitat loss and alteration [51] or climate change and pollution [52], on native taxa and ecosystem functioning. This seems particularly important for alien taxa that have been intentionally introduced to restore ecological functions [53,54] or to act as biological control agents against native or alien pests [55]. Similar considerations can be made for thermophilic alien species securing ecological functions that would have been lost otherwise due to sea warming–induced declines or extinctions of key native species, as, for example, in the eastern Mediterranean Sea [56,57].

To fill this current gap, in this Consensus View, we propose a framework for assessing the degree to which alien taxa positively affect native biodiversity: the positive Environmental Impact Classification for Alien Taxa, or EICAT+. The framework structurally resembles EICAT, using 5 semiquantitative scenarios to describe the magnitude of observed positive impacts of alien taxa on native biodiversity attributes (specifically, increases in individual performance, population size, and area of occupancy). In EICAT+, the highest levels of impact magnitude are assigned to alien taxa increasing the area of occupancy of native taxa through local population reestablishment or preventing local extinction. The framework also describes the underlying impact mechanisms by which alien taxa can increase native biodiversity attributes, such as through food and habitat provision, dispersal facilitation, or disease reduction. To illustrate the functionality and utility of the proposed framework, we apply EICAT+ to case studies reporting positive impacts of alien taxa on native biodiversity across different taxonomic groups and ecosystems. Advantages and applications, as well as limits and potential misuses of the scheme, are also discussed.

## Development process and structure of EICAT+

The development of EICAT+ involved a 4-stage process of expert elicitation. At stage 1, a core panel of experts who had previously contributed to develop and improve EICAT [10,24,25,61] and were familiar with its application across various invasion contexts formulated a preliminary version of EICAT+. At stage 2, multiple experts from the invasion science community, many of which contributed to the development of the EICAT framework [10], were contacted and asked to provide input and feedback toward the core concepts of the framework under development. These experts were selected due to their extensive knowledge regarding the impacts of alien species and mechanisms through which these impacts are generated. At stage 3, and once a general consensus was reached between the experts, the core panel led the writing of the first and subsequent drafts of a manuscript regarding EICAT+. Several versions of the manuscript were produced, and, at each round, the experts involved were asked to provide substantial input into ideas, text, and figures. Importantly, experts also provided and reviewed real-world examples of positive impacts caused by alien species based on their taxonomic and ecological expertise; such examples were critical for testing the applicability of EICAT+ across various invasion contexts (see Supporting information D and E in S1 File). At stage 4, the final version of the framework and related manuscript were finalized and approved by all experts involved in this Consensus View.

**Fig 2:**
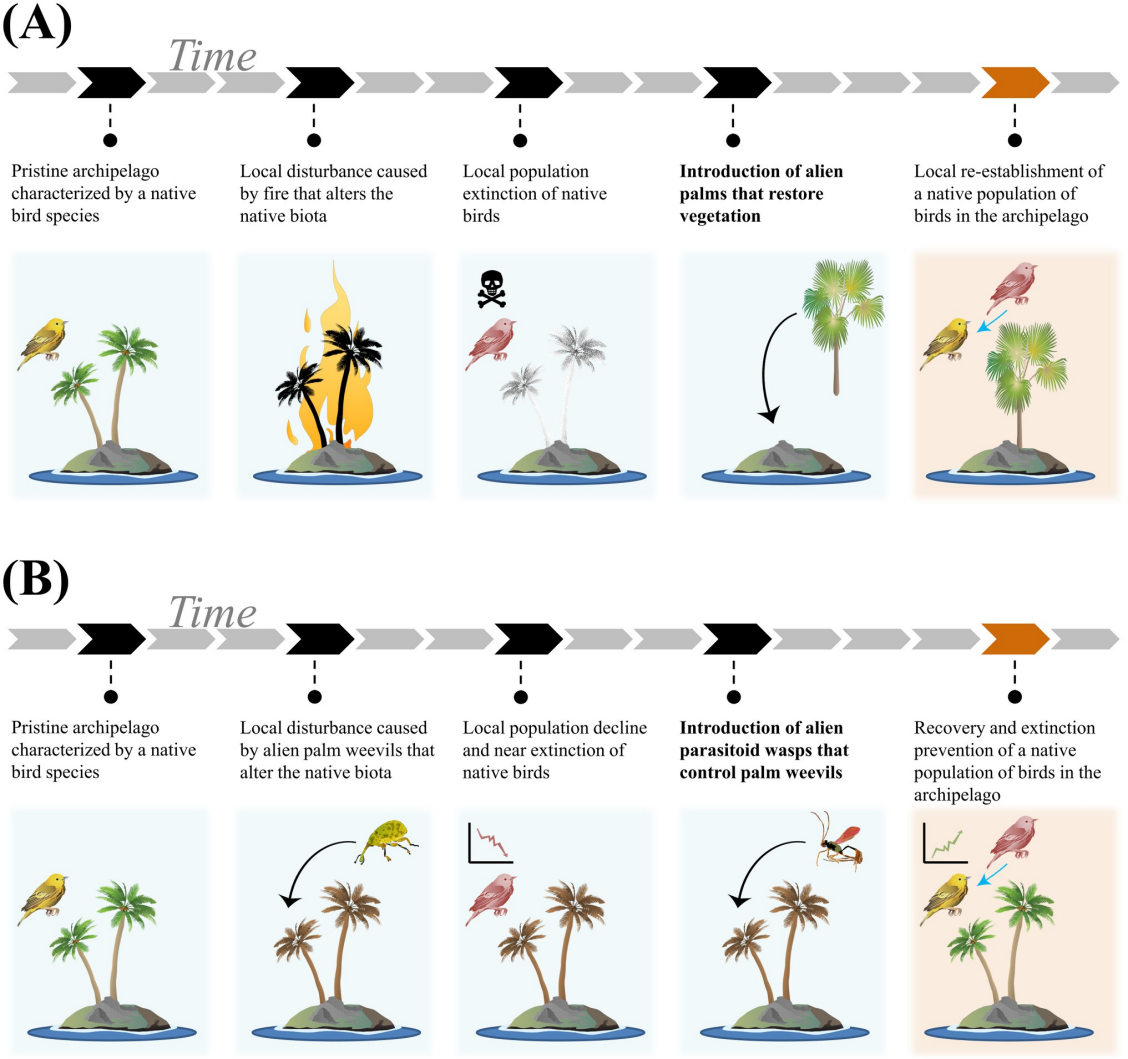
Examples of Major positive impacts under EICAT+. Hypothetical examples of Major positive impacts (MR+) caused by an alien palm (A) and an alien parasitoid wasp (B) on a local population of a native bird species (taxon of interest) on an archipelago. (A) The alien palm causes the local reestablishment of the native bird species, e.g., via natural dispersal of birds across the archipelago. (B) The alien parasitoid wasp acts as a classical biocontrol agent against alien palm weevils and prevents the extinction of the bird population. Note that under EICAT+, the impact is classified as Major regardless of whether the palm weevil is alien or native (see also submechanisms 10.1 and 10.2 in Fig 3), as the indirect positive impact is caused by an alien taxon (the parasitoid wasp). Since it can be assumed that the newly established population (A) or the recovered population (B) would not persist if the alien taxon causing the positive impact was no longer present on the island, the impacts should not be classified as Massive (MV+), i.e., the alien palm or wasp must continue to be present on the island for the native bird species to survive. Black arrows indicate introduction and establishment of alien taxa into a recipient ecosystem. Blue arrows indicate the reestablishment (A) and extinction prevention (B) of a native taxon due to an alien taxon. Symbols were courtesy of the Integration and Application Network, University of Maryland Center for Environmental Science (ian.umces.edu/media-library).

**Fig 3:**
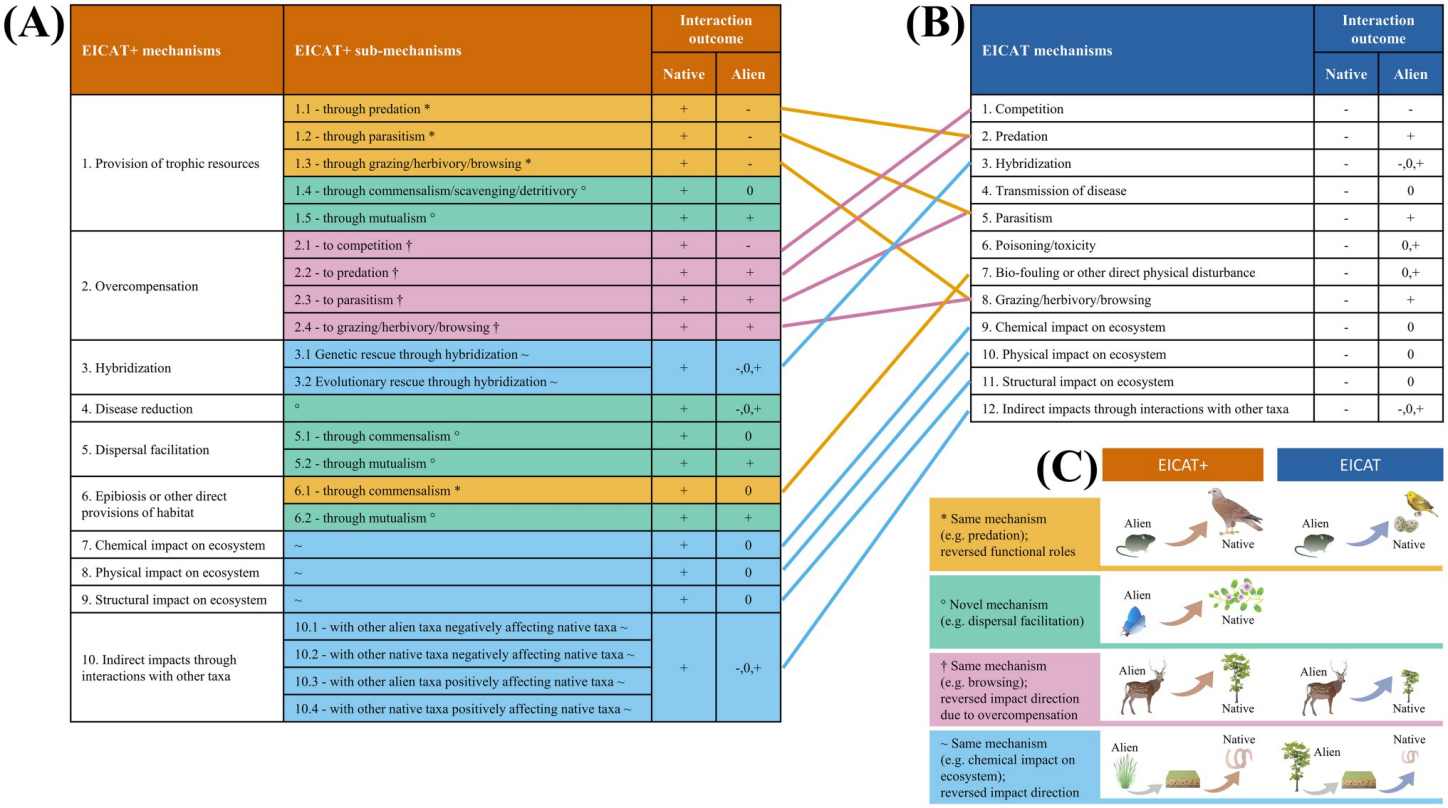
EICAT+ and EICAT mechanisms and submechanisms. List of EICAT+ mechanisms and submechanisms (A) and EICAT mechanisms (B). EICAT+ and EICAT mechanisms and submechanisms are also compared to each other (C) based on the outcome of the interaction for native and alien taxa. Colors of rows and connecting lines reflect different rationales behind the formulation of the EICAT+ mechanisms and submechanisms, with the different colors and symbols that indicate [yellow and asterisks “*”] mechanisms present in both EICAT+ and EICAT but in which the functional roles of alien and native taxa are reversed (e.g., in EICAT+, the alien taxon is the prey, whereas in EICAT, the alien is the predator); [green and degree signs “°”] mechanisms that are unique to EICAT+ (e.g., dispersal facilitation through pollination); [pink and daggers “†”] mechanisms present in both EICAT+ and EICAT but in which impact direction is reversed because of overcompensation (e.g., in EICAT+, the alien taxon increases growth of the native taxon through browsing-mediated overcompensation, whereas in EICAT, the alien taxon decreases growth of the native taxon through browsing; [blue and tildes “~”] mechanisms present in both EICAT+ and EICAT but in which impact direction is reversed (e.g., in EICAT, the alien taxon decreases a biodiversity attribute by impacting the chemistry of the ecosystem, and in EICAT+, the alien taxon increases a biodiversity attribute by impacting the chemistry of the ecosystem). The symbols +, −, 0 indicate positive, negative, and neutral outcomes of interactions between a native and an alien taxon. In C, arrows indicate impacts of an alien to a native taxon (orange arrow: positive impact; blue arrow: negative impact). Symbols were courtesy of the Integration and Application Network, University of Maryland Center for Environmental Science (ian.umces.edu/media-library).

Overall, we have structured the novel framework to align with EICAT using semiquantitative scenarios to assign an alien taxon to one of 5 ascending categories of impact magnitude. The EICAT+ scenarios describe how alien taxa can increase native biodiversity attributes at different levels of biological organization. The scenarios are thus hierarchically organized to describe all observed positive impacts. In accordance with EICAT, EICAT+ (a) considers impacts at the level of individuals (Minimal and Minor) and populations (Moderate, Major, and Massive) of a native taxon (Fig 1); (b) distinguishes between Major and Massive impacts on the basis of the reversibility of an impact after the disappearance of the alien taxon from its recipient system (Fig 2); (c) assigns a confidence score for each recorded impact to provide an estimate of uncertainty; (d) describes the different mechanisms by which alien taxa can impact native individuals and populations (Fig 3); and (e) performs the global assessment of a taxon by collecting evidence of impact from the taxon’s entire alien range and classifying the taxon based on the highest criterion level met anywhere in the alien range and across any of the impact mechanisms [24]. It should be noted that an alien taxon can be assessed under EICAT+ as having a positive impact on 1 native taxon and under EICAT as having a negative impact on another native taxon or different impacts on the same taxon but at different locations or in different contexts.

### Magnitude of positive impacts

The 5 scenarios used in EICAT+ to assign alien taxa to impact categories structurally resemble those reported in EICAT (Table 1 and Fig 1).

**Table 1:** Scenarios used in EICAT and EICAT+ to assign alien taxa to impact categories. Detailed description of the 5 semiquantitative scenarios used in EICAT [24] and EICAT+ to assess negative and positive impacts caused by alien taxa on native taxa at different levels of organization. The scenarios are used in EICAT and EICAT+ to assign alien taxa to one of 5 ascending categories of impact magnitude. Note that when there is no or inadequate information to classify an alien taxon to one of the 5 impact categories, the taxon should be classified as Data Deficient (DD).

EICAT (Environmental Impact Classification for Alien Taxa)	EICAT+ (positive Environmental Impact Classification for Alien Taxa)
**Minimal Concern (MC)** A taxon is considered to have impacts of Minimal Concern when it causes negligible levels of impacts, but no reduction in performance of individuals in the native biota. Note that all alien taxa have impacts on the recipient environment at some level, for example, by altering species diversity or community similarity (e.g., biotic homogenization), and for this reason, there is no category equating to “no impact.” Only taxa for which changes in the individual performance of natives have been studied but not detected are assigned an MC category. Taxa that have been evaluated under the EICAT process but for which impacts have not been assessed in any study should not be classified in this category, but rather should be classified as Data Deficient.	**Minimal positive impact (ML+)** A taxon is considered to have Minimal positive impacts when it interacts with at least 1 native taxon through a mechanism that can lead to positive impacts but causes only a negligible increase in the performance of individuals of the native taxon. Note that although alien taxa are often thought to cause positive impacts on native taxa—for instance, by serving as a food source for native consumers—these impacts should be considered minimal if the alien simply fulfills a functional role already held by other taxa without increasing the performance of individuals of the native taxon. Only alien taxa for which positive changes in the individual performance of native taxa have been studied but not detected are assigned an ML+ category. Alien taxa that have been evaluated under the EICAT+ process but for which impacts have not been assessed in any study should not be classified in this category, but rather should be classified as Data Deficient.
**Minor impact (MN)** A taxon is considered to have Minor impacts when it causes reductions in the performance of individuals in the native biota, but no declines in native population sizes, and has no impacts that would cause it to be classified in a higher impact category.	**Minor positive impact (MN+)** A taxon is considered to have Minor positive impacts when it causes increases (or prevents/mitigates decreases) in the performance of individuals of at least 1 native taxon, but no increases in native population sizes, and has no positive impacts that would cause it to be classified in a higher impact category.
**Moderate impact (MO)** A taxon is considered to have Moderate impacts when it causes declines in the population size of at least 1 native taxon, but has not been observed to lead to local extinction of a native taxon.	**Moderate positive impact (MO+)** A taxon is considered to have Moderate positive impacts when it causes increases (or prevents/mitigates decreases) in the population size of at least 1 native taxon but has not been observed to promote local population reestablishment, or to prevent local population extinction, of a native taxon.
**Major impact (MR)** A taxon is considered to have Major impacts when it causes community changes through the local or subpopulation extinction (or presumed extinction) of at least 1 native taxon, which would be reversible if the alien taxon was no longer present. Its impacts do not lead to irreversible local population, subpopulation, or global taxon extinctions.	**Major positive impact (MR+)** A taxon is considered to have Major positive impacts when it causes transient increases (or prevents transient decreases) in species occupancy through local or subpopulation reestablishment (or extinction prevention) of at least 1 native taxon, which would be reversed if the alien taxon was no longer present.
**Massive impact (MV)** A taxon is considered to have Massive impacts when it causes irreversible community changes through local, subpopulation, or global extinction (or presumed extinction) of at least 1 native taxon.	**Massive positive impact (MV+)** A taxon is considered to have Massive positive impacts when it causes long-lasting increases (or prevents long-lasting decreases) in species occupancy through local or subpopulation reestablishment (or extinction prevention) of at least 1 native taxon, which would remain even if the alien taxon was no longer present.

The first EICAT+ scenario, Minimal positive impact (ML+), describes negligible increases in the individual performance of native taxa. Minimal positive impacts describe cases in which the alien and native interact through one of the mechanisms that can potentially lead to a positive impact (Fig 3), but no increase in performance (or higher-level impact) has been detected. This may occur when alien taxa simply fulfill the same functional role of other taxa without increasing the performance of native individuals [35]. For instance, alien plants can provide pollen and nectar to native pollinators [42,62,63], or alien fish can become a food source for native predatory fish [64,65], but there is no detectable evidence that these interactions improve the survival, growth, or body size of the native consumers. Note that taxa classified as Minimal positive under EICAT+ should not be confused with those classified as Data Deficient (DD), i.e., when no information is available to measure an impact or there are methodological issues with the evidence available such that it is not sufficiently reliable to classify the positive impact magnitude (see dedicated paragraph below). Note also that taxa classified as Minimal positive under EICAT+ should also not be confused with those classified as Minimal Concern under EICAT, i.e., when the alien and native taxa interact through one of the mechanisms that can lead to a negative impact (e.g., competition; Fig 3), but no decrease in the performance of the native taxa has been detected (Table 1 and Fig 1).

The second EICAT+ scenario, Minor positive impacts (MN+), describes increases in the performance of native individuals caused by alien taxa (Table 1 and Fig 1), such as increases in rates of growth, fecundity, photosynthesis, or hunting success of the native organisms, but no increase in the population size of the native taxon, due to the alien taxon, is detected. Examples of MN+ impacts include the following: an alien plant increasing the growth rate of a native plant (here the taxon of interest) through the production of allelochemicals suppressing pathogenic fungi [66]; an alien plant improving the hunting success of native spiders by providing foraging habitats [67]; an alien fish decreasing the abundance of a competitively dominant native fish, thus indirectly increasing the juvenile growth rate of competitively subordinate native fish [68] (here the entity of interest); or an alien plant increasing pollen availability in a native plant by attracting pollinators to the location (magnet species effect), thus increasing the reproductive success of the native plant [69].

The third EICAT+ scenario, Moderate positive impacts (MO+), describes cases in which an alien taxon increases the population size of at least 1 native taxon (or prevents/mitigates an ongoing population decline) (Table 1 and Fig 1). MO+ examples include the following: increases in the abundance of native frugivorous birds due to the provision of fleshy fruits from alien shrubs [70]; increases in the abundance of a native mayfly due to epibiosis on the surface of an alien bivalve [71]; increases in the abundance of mussels due to enhanced recruitment and survival caused by an alien green alga through the creation of a novel microhabitat on breakwaters [72]; or increases in the abundance of multiple native ground-dwelling invertebrates from different feeding guilds in the litter under alien plants [73].

The fourth and fifth EICAT+ scenarios, Major positive impacts (MR+) and Massive positive impacts (MV+), respectively, refer to increases in species occupancy of at least 1 native taxon through local reestablishment of native populations (or extinction prevention; see Supporting information C in S1 File) within their native ranges (Table 1 and Fig 1). Alien taxa may contribute to native taxa reestablishment that could not have occurred in their absence. To be considered in EICAT+, such reestablishment may occur via natural colonization or reintroduction by humans (either accidental or deliberate). Reintroductions that lead to reestablishment will be considered in EICAT+ only if no additional conservation effort to reintroduce native individuals has been made since the introduction of the alien taxon, i.e., if reintroductions occurred at rates similar to those before the alien was introduced [24] and if there is a clear evidence that reestablishment would not be successful in the absence of the alien taxon (i.e., any extra conservation effort to recolonize would be necessary but not sufficient). Impacts are not classified in EICAT+ when an alien taxon renders areas outside the native range of the native taxon suitable to the latter, e.g., when the alien would allow a species to expand beyond its range (a so-called “neonative” [74]; Supporting information A in S1 File). The distinction between Major and Massive impacts for EICAT+ is in their reversibility. When evidence indicates that the reestablished native population, or the native population whose extinction was prevented, would go extinct if the alien taxon was no longer present, the positive impact is only transient and would be classified as Major (MR+). If the reestablished native population, or the native population whose extinction was prevented, would persist even if the alien taxon was no longer present, the positive impact is long lasting and would be classified as Massive (MV+). Because all populations go extinct eventually and population trajectories can only be reliably forecasted for the near future, positive impacts are classified as Massive only if the reestablished native population, or the native population whose extinction was prevented, would persist for at least 10 years or 3 generations (whichever is longer) after the disappearance/removal of the alien taxon. Such practical time intervals are analogous to the intervals chosen in EICAT to judge the reversibility of local extinctions caused by the alien and distinguish between Major and Massive negative impacts [24].

Conceptual examples of Major positive impacts promoted by an alien taxon through local reestablishment or extinction prevention can be found in Fig 2. Cases reported in the literature are as follows: the return of a native bird into areas within its native range from which it temporarily disappeared and where the bird now uses fledging sites provided by an alien plant [75] or the reestablishment of native plants caused by changes to the chemical, physical, and structural soil properties caused by an alien tree [51]. Additionally, alien taxa might prevent the extinction of native populations that were observed to be in decline before the introduction of the alien taxon. MR+ examples in which alien taxa might have prevented the extinction of native species include the following: the provision of roosting sites by an alien tree to a native butterfly that lost its historical roosting sites due to logging and habitat alteration [62]; the provision of refugia by an alien plant to 3 critically endangered and protected native snails suffering from alien rat predation [50]; or the mutualism established by alien zooxanthellae with bleached native corals, thus preventing the disappearance of photosynthetic corals under climate change–induced thermal stress [76,77].

Massive positive impacts (MV+) can be caused by the demographic rescue effect (i.e., populations persist due to a boost in population size [78]) that an alien producer might have on the population of a native consumer previously trapped in an extinction vortex. Other real-world examples can include the following: the long-lasting impact that an alien biocontrol beetle had on a declining endemic plant by eradicating an alien scale pest [79,80]; and the genetic or evolutionary rescue induced by alien individuals on native populations through hybridization [81,82]. In these examples, the positive impacts of the alien taxa on the native populations would persist even if the alien taxa are removed or disappear naturally from the area.

Note that as in EICAT, if there is no or inadequate information to classify an alien taxon to one of the 5 categories of impact magnitude, the taxon should be classified as DD [24,25]. As a consequence, studies reporting mechanisms by which an alien taxon may cause positive impacts on native taxa, but that do not measure, or provide information on, changes in biodiversity attributes relevant to EICAT+ (e.g., performance of individuals), do not contribute information that would allow an impact to be scored, implying a DD classification. However, we strongly recommend that when available such information still be reported (e.g., in supplementary materials), to document research gaps which might inspire future studies around positive impacts of alien species (see Supporting information E in S1 File).

Analogous to EICAT, EICAT+ assigns confidence scores (high, medium, low) to each impact record depending on how confident the assessor is that the assigned impact magnitude reflects the true situation [25,83]. A general discussion about why and how to account for uncertainty when assessing alien species impacts can be found in Probert and colleagues [83], while more detailed explanations of how to assign confidence can be found in the Supporting information F in S1 File.

### Mechanisms of positive impacts

The mechanisms through which alien taxa increase native biodiversity attributes are grouped similarly to the mechanisms described in EICAT [24,25], facilitating high consistency and comparability between both assessment schemes. Note that not all mechanisms that occur in EICAT also exist in EICAT+; positive impacts may also be caused through facilitative interactions [35,37,39,84,85] that have no negative equivalent in EICAT. In Fig 3, we provide a list of mechanisms (and submechanisms) describing all interactions through which an alien taxon (A) can positively impact a native taxon (N). The alien taxon itself can either be positively (mutualistic interactions; N+/A+), negatively (antagonistic interaction N+/A−), or neutrally (commensalistic interactions; N+/A0) impacted by such interactions. Additional details around the rationale behind the formulation of the EICAT+ mechanisms and submechanisms can be found in Fig 3 and Supporting information G in S1 File. Real-world examples of positive impacts caused by alien taxa through different EICAT+ mechanisms and submechanisms can be found in the Supporting information D and E in S1 File.

## Practicality of EICAT+

Comprehensive and holistic understanding of the multiple changes caused by alien taxa on native biodiversity requires considering and quantifying both negative and positive impacts. However, to date, no standardized and evidence-based framework has been available to assess positive impacts [8]. EICAT+ provides such a framework, and, together with the global standard EICAT, can help improve theoretical and practical understanding on whether, and to what degree, alien taxa positively and negatively impact native taxa and to translate such improved knowledge into practical solutions. Since the framework can be applied to all taxonomic groups, and at different spatial scales, from local to global, EICAT+ allows objective comparisons across taxa and regions. For instance, EICAT+ can inform efforts to restore native biodiversity that historically characterized certain protected areas by ranking taxonomically diverse alien taxa in accordance with their positive impacts, so that taxa found to have minimal positive impacts (through EICAT+) but harmful impacts (through EICAT) can be targeted for removal without causing “unforeseen” negative consequences on native biodiversity. Alternatively, EICAT+ can be used to rank closely related alien taxa across regions and habitats, such as insect species introduced for biological control, so that taxa causing the highest positive impacts on native biodiversity are transparently and rigorously identified. Rank orders obtained through comparisons based on EICAT+ might also be tested against life history traits, climate variables, or habitat types, to investigate factors correlating with magnitude and type of positive impacts (for analogous investigations on negative impacts, see, for instance, Evans and colleagues [30] and Kesner and Kumschick [86]). Below, we identify some specific cases in which the application of EICAT+ could generate practical recommendations and support evidence-based management decisions for nature conservation and restoration. In addition, we explore the limits of EICAT+ and briefly discuss under which conditions the scheme should (and should not) be used.

### EICAT+ helps to forecast unwanted consequences of alien taxa control

Alien taxa are often ranked in priority lists according to the degree to which they harm certain entities of interest such as species or ecosystems. Such lists can be used to inform which species should be prevented, contained, or eradicated and how management efforts should be allocated to mitigate deleterious impacts [87]. Often, however, taxa that are prioritized for management because of their negative impacts, and which might be perceived as unwanted, also cause positive impacts, which might be perceived as favorable (Fig 1) (see also Milanović and colleagues [88]). For instance, in a review of impacts by alien marine taxa on biodiversity and ecosystem services, out of 87 high-impact taxa, only 17 had exclusively negative and 7 exclusively positive impacts, whereas 63 had both negative and positive impacts [5]. High-impact taxa can also have negative effects on biodiversity within 1 trophic level, e.g., through antagonistic interactions (competition and predation), but positive effects on higher trophic levels, e.g., through habitat and food provisioning [89,90] or predator release [91]. Given such multidirectional impacts, the EICAT+ assessment will have practical utility (especially when used in combination with EICAT) for managers, policymakers, and the public, as it allows forecasting whether, and how, alien taxa management may yield unwanted consequences for native biodiversity [47]. For example, the eradication of alien cats (*Felis catus*) on Macquarie Island in the South Pacific to lessen their predation pressure on native seabirds led to an increase in the abundance of alien rabbits (*Oryctolagus cuniculus*) and, consequently, caused the local disappearance of native large-leaved herbs and grasses [92]. Similarly, the removal of alien trees, *Casuarina* spp., on the Japanese Ogasawara Islands led to the local disappearance of endangered snail species that found refugia from predatory alien rats (*Rattus rattus*) in the *Casuarina* litter [50]. In both cases, it would have been useful to promptly report and systematically assess indirect positive impacts caused by the target species so that countermeasures could have been implemented to avoid or mitigate undesirable outcomes.

More generally, an EICAT+ assessment will provide stakeholders (including managers, policymakers, and the public) with evidence-based information that can guide the implementation of adequate countermeasures and avoid or minimize undesirable outcomes in 2 principal ways. First, the benefits of removing a certain deleterious alien species can be compared to the costs that the removal imposes on other native species [49,54,93,94]. The necessity of conducting such a cost–benefit analysis has been recently stressed by Rees and colleagues [95], who suggested that the choice of removing dingoes (*Canis lupus dingo*) in some parts of Australia should be assessed case by case, as these apex predators increase the abundance of native small mammal species and a native owl species by suppressing 2 alien mesopredators (*Vulpes vulpes* and *F*. *catus*). Similar considerations have been made by Gomes and colleagues [96], who noted the need for careful evaluation of management decisions regarding alien jackfruit trees (*Artocarpus heterophyllus*) in a secondary forest of Brazil, where these plants contribute to the maintenance of frugivore populations and promote seed rain and seedlings of native plants. Second, the identification of alien taxa that mitigate the harmful impacts of other alien taxa can be used to design adaptive management strategies with a specific removal order. In the above examples, the removal of alien cats and *Casuarina* trees should be preceded by, or carried out in tandem with, the removal of alien rabbits [92] and rats [50], respectively. Mapping alien–alien antagonistic interactions and their effect on native taxa has been proven to be useful in several cases, for instance, in designing an adaptive eradication program for alien rats on a tropical Pacific island [97]. Here, since data from different trophic levels showed that the rats competed with alien mice and consumed an alien plant species, rats and mice were removed simultaneously and only after the alien plant eradication, so that unwanted cascading effects on the insular ecosystem could be prevented.

### EICAT+ helps to assess functional restoration and biological control

Only a subset of alien animals, plants, and microorganisms cause severe harmful impacts on biodiversity [7,98], and while many alien taxa are inconsequential, some might even facilitate restoration. Many populations have disappeared, or are currently heading toward extinction, within their native range as a consequence of various anthropogenic stressors such as climate change, alien taxa, pollution, habitat loss, and habitat degradation [99,100]. Certain species have been deliberately introduced to restore lost ecological functions, while others have been tolerated due to their capacity to counterbalance or mitigate environmental stressors or disturbance. Examples are as follows: an alien tortoise species (*Aldabrachelys gigantea*) introduced to the Mascarene Islands east of Madagascar to restore seed-dispersal functions of recently extinct species promoted seedling patches of endangered trees through endozoochory [101]; males from a subspecies of boobook owl (*Ninox novaeseelandiae novaeseelandiae*) introduced to Norfolk Island rescued another subspecies of boobook owl from extinction [82]; or an alien oyster (*Crassostrea gigas*) increased water clarity, provided substrate and shelter, and acted as a food source for native species in deteriorated and heavily modified ecosystems in the Netherlands [102]. In addition, the use of alien taxa as classical biological control agents to limit or suppress introduced weeds and pests and protect native biodiversity has been carried out on multiple occasions [55,103]. For instance, an alien predatory lady beetle (*Hyperaspis pantherine*) was used in successful biological control of an alien scale insect on St. Helena Island in the South Atlantic, thus preventing the extinction of a native endemic gumwood tree [80]. Analogously, the introduction of an alien weevil (*Cyrtobagous salviniae*) has been advocated to suppress an alien floating aquatic fern and, therefore, to promote the recovery of native aquatic algae and macroinvertebrates in South Africa [104]. Each biocontrol release requires extensive research on host specificity, a comprehensive evaluation of potential and actual impacts, and post-release monitoring to track the impacts (both positive and negative) [105]. EICAT+ promises to be a powerful and transparent means to quantify to what degree restoration and biocontrol programs based on alien species offer positive outcomes to native biodiversity conservation. Quantifying positive impacts will be instrumental in indicating which of these management programs should be maintained or enhanced and on the contrary, which should be revised or even avoided in the future. Alien taxa used in restoration and biocontrol that score high with EICAT+ (i.e., having positive impacts at the population level that can be classified as Moderate, or higher), and without relevant negative impacts, could also be subjected to specific conservation actions (for instance, see the mitigation strategies proposed to reduce the effects of climate change on rewilded populations of tortoises [106]), so that their positive impacts on native biodiversity are maintained in the future.

### EICAT+ facilitates the distinction between real and unproven positive impacts

As opposed to negative impacts, positive impacts of alien species are claimed to often be described only anecdotally or not rigorously [20,107]. In many cases, the literature describes the existence of mechanisms by which alien taxa might cause positive impacts, for instance, by acting as a food source for native taxa [108,109], but fails (or avoids) measuring the actual consequences they have on native taxa (Supporting information E in S1 File). EICAT+ identifies such information gaps and flags the need to explicitly measure relevant biodiversity attributes (e.g., population size) so that inconsequential impacts (e.g., Minimal positive) and relevant, but currently unreported impacts (e.g., Minor positive, or higher), can be distinguished. A lack of in-depth knowledge may result in positive impacts being overlooked, and also in the erroneous classification of negative impacts as positive. For example, laboratory trials and field surveys showed that a native trout species readily consumed an alien New Zealand snail species in North America, although individuals feeding upon alien snails had lower growth and body condition than those which did not consume any alien snails [110]. Similarly, multiple native butterflies lay their eggs on alien plants in the US, although their larvae rapidly die because of the toxicity of the plants [58]. Although at first glance the impacts above seemed positive, they turned out to have negative consequences for native taxa due to eco-evolutionary traps, i.e., maladaptive responses made regardless of the availability of better options [111,112]. These examples indicate that positive impacts should not be automatically inferred from the mechanisms—as the latter could underlie negative impacts that can be assessed under EICAT, not EICAT+. Therefore, EICAT+ can be a useful guide to identify true positive impacts.

### EICAT+ is not a framework to assess value-laden beneficial impacts and should not be used to offset or understate negative impacts

While it may be tempting to discount negative EICAT with positive EICAT+ scores for an alien taxon in an attempt to “offset” negative impacts with positive ones, this would be simplistic and even misleading for management decisions. Positive impacts assessed under EICAT+ should not be confused with beneficial impacts, i.e., impacts that are perceived as favorable or desirable in accordance with certain values and motivations (Box 1). Indeed, a large part of the positive impacts assessed under EICAT+ can be seen by some as harmful, for instance, by those who consider all changes caused by an alien taxon to the recipient ecosystem as unwanted alterations of its state. Under this conservation standpoint, an alien shrub species that simultaneously decreases and increases the population size in a native grass species and in a native bird species, respectively (as in Fig 1), would be considered harmful in both cases. Moreover, many positive impacts caused by alien taxa on native taxa can have cascading harmful effects on protected native taxa or human communities. For instance, local reestablishment of a native predatory bird favored by an alien taxon might put additional pressures on endangered native prey species, while increases in the abundance of a dominant native grass species might threaten rare subordinate species. Increases in the population size of a native weed species can hamper agricultural productivity or lead to acute allergic reactions, while deleterious consequences for human well-being can also be expected when native pests, such as wild boar, mosquitoes, or bark beetles, become more abundant. In the above examples, EICAT+ would assess the positive impacts of alien taxa on the native predatory bird, grass, weed, or pest species with value-free criteria, i.e., without evaluating whether such impacts are favorable or unfavorable from a certain perspective or for different stakeholders [8]. Such evaluation can be undertaken at a later stage by weighting or selecting EICAT+ data in accordance with specific values, motivations, or perceptions [113]. For instance, the conceptual framework proposed by Kumschick and colleagues [9], which combines objectively measured impacts with their importance for affected stakeholders, was developed to produce weighted impact scores and facilitate prioritization. Value-weighted scores can also help to identify societal or nature conservation conflicts associated with alien taxa [114,115] and inform risk analysis [31] or management decisions [8,116]. Future research should explore ways to link people’s perceptions with impact assessments generated under both EICAT and EICAT+, so that changes caused by alien taxa can be understood in their full complexity.

## Concluding remarks

In this Consensus View, we presented EICAT+, a framework to assess positive impacts caused by alien taxa on native biodiversity. The framework can be applied to all alien taxa, from plants to animals, fungi, and even microorganisms, and at different spatial and organizational scales. EICAT+ fills a critical gap in the invasion science community, as it provides a standardized and evidence-based framework for the assessment of positive impacts that was not available before. While relying on the premise that a distinction between native and alien taxa is well grounded in conservation [11], EICAT+ recognizes that certain alien taxa increase native biodiversity attributes and provides an objective scientific base to evaluate how these taxa may contribute to conservation objectives [36]. When used alone, or in combination with other assessment schemes such as EICAT, the IUCN global standard for reporting the negative impacts of alien taxa, EICAT+ will provide systematic and transparent data regarding the multiple changes caused by alien taxa on native taxa. Structured assessment of all impacts on native biodiversity can enrich our understanding of the consequences of biological invasions and improve nature conservation.

## Supporting information

S1 File Supporting information. **Supporting information A in S1 File. Glossary of additional key terms. Supporting information B in S1 File. Table reporting contrasting arguments and approaches used to define how alien taxa are considered and should be managed in accordance with different conservation values/motivations.** As multiple values/motivations exist and determine which entities we are interested in (see also Supporting information A), distinct conservation targets can be identified. Note that here, we only consider conservation values/motivations that are expressed regardless of any nature’s instrumental (utilitarian) value, i.e., regardless of nature’s contributions to human well-being (see “nature for itself” framing [9]). Also, note that such contrasting arguments and approaches are not necessarily mutually exclusive and have been occasionally combined to find a middle ground to achieve broader conservation goals [10–13]. **Supporting information C in S1 File. Circumstances under which the prevention/mitigation of a decreasing change is considered as a positive change under EICAT+.** In EICAT+, we also consider as positive impacts (i.e., increasing changes) cases in which an alien species prevents/mitigates decreasing changes, e.g., when the performance of a native individual, the size of a native population, or the occupancy of a native species would have decreased, or decreased to a greater extent, if the alien species had not been introduced. Although some of these positive impacts can be inferred, the prevention of a decreasing change should be assessed under EICAT+ only when there is convincing evidence that a certain biodiversity attribute (e.g., population size) would have decreased, or decreased to a greater extent, in the absence of the alien species. In the case of extinction prevention, for instance, it must be clear that (i) the population was locally heading toward extinction before the introduction of the alien; and (ii) the alien taxon prevented, through a specific impact mechanism, an extinction that would have occurred in its absence [41,42] (Fig 2b). Other cases where an alien species may prevent or mitigate decreasing changes are, for instance, those in which the abundance (i.e., a proxy for population size) of a native species declined in the uninvaded (i.e., control) plots but not, or to a lesser extent, in the plots invaded by the alien. Note that positive impacts associated with the prevention/mitigation of a decreasing change will generally be more difficult to study and identify than those associated with actual increasing changes, as the former require extensive data regarding the temporal trend of individual performance, population size, or area of occupancy. **Supporting information D in S1 File. EICAT+ mechanisms and submechanisms by which an alien taxon can cause positive impacts on native biodiversity attributes and examples of positive impacts sourced from the literature and assessed under EICAT+** (ML+ = Minimal positive impact, MN+ = Minor positive impact, MO+ = Moderate positive impact, MR+ = Major positive impact, MV+ = Massive positive impact). Rationales behind the formulation of the mechanisms and submechanisms can be found in the main text and in Supporting information G, H, and J. **Supporting information E in S1 File. Table reporting examples sourced from the literature and classified as information that cannot be classified under EICAT+, but that contain information about mechanisms and might set the stage for future studies.** Although these studies described the existence of mechanisms by which alien taxa may cause positive impacts on native taxa, such literature is considered as nonrelevant, as it did not measure, or provide information on, biodiversity attributes used in EICAT+ (e.g., performance of individuals or population size). Rationales behind the formulation of the mechanisms and submechanisms can be found in the main text and in Supporting information G, H, and J. **Supporting information F in S1 File. How to attribute a confidence score in EICAT+. Supporting information G in S1 File. Additional information around the rationale behind the formulation of the EICAT+ mechanisms and submechanisms. Supporting information H in S1 File. Additional information about how alien species can cause positive impacts on native biodiversity through overcompensation. Supporting information J in S1 File. Additional information about how alien species can cause positive impacts on native biodiversity through hybridization. Supporting information K in S1 File. References used in the Supporting information.**
(DOCX)

## Abbreviations

DD: Data Deficient
EICAT: Environmental Impact Classification for Alien Taxa
EICAT+: positive Environmental Impact Classification for Alien Taxa
IUCN: International Union for Conservation of Nature
