# Supplementary material for: The EICAT+ framework enables classification of positive impacts of alien taxa on native biodiversity
Source: PLoS Biol. 2022 Aug 16;20(8):e3001729. doi: 10.1371/journal.pbio.3001729 (PMC9380921; doi:10.1371/journal.pbio.3001729)
Supplement: S1 File — Supporting information A in S1 File. Glossary of additional key terms. Supporting information B in S1 File. Table reporting contrasting arguments and approaches used to define how alien taxa are considered and should be managed in accordance with different conservation values/motivations. As multiple values/motivations exist and determine which entities we are interested in (see also Supporting information A), distinct conservation targets can be identified. Note that here, we only consider conservation values/motivations that are expressed regardless of any nature’s instrumental (utilitarian) value, i.e., regardless of nature’s contributions to human well-being (see “nature for itself” framing [9]). Also, note that such contrasting arguments and approaches are not necessarily mutually exclusive and have been occasionally combined to find a middle ground to achieve broader conservation goals [10–13]. Supporting information C in S1 File. Circumstances under which the prevention/mitigation of a decreasing change is considered as a positive change under EICAT+. In EICAT+, we also consider as positive impacts (i.e., increasing changes) cases in which an alien species prevents/mitigates decreasing changes, e.g., when the performance of a native individual, the size of a native population, or the occupancy of a native species would have decreased, or decreased to a greater extent, if the alien species had not been introduced. Although some of these positive impacts can be inferred, the prevention of a decreasing change should be assessed under EICAT+ only when there is convincing evidence that a certain biodiversity attribute (e.g., population size) would have decreased, or decreased to a greater extent, in the absence of the alien species. In the case of extinction prevention, for instance, it must be clear that (i) the population was locally heading toward extinction before the introduction of the alien; and (ii) the alien taxon prevented, through a specific impact mech [file pbio.3001729.s001.docx]

**Supporting information**

The EICAT+ framework enables classification of positive impacts of alien taxa on native biodiversity

| **Supporting information A - Glossary of additional key terms**  **Entity of interest:** an entity, such as a population or a habitat, that is relevant to people according to certain values, motivations and reference systems and can benefit or suffer from the impact of an alien taxon. Entities of interest can be, for instance, native species, sentient individuals, protected populations or ecosystems, human communities and societies. Ethical, societal or conservational values are crucial to define not only the entity of interest but also to what extent the latter benefits or suffers from the impact of an alien species. For example, supposing that an alien predator decreases the population size of a native prey species, the impact can be considered beneficial for ecosystem functioning if the native species has become, for various reasons, overabundant and competes strongly with subordinate species, or harmful to nature conservation if the native species is rare, protected or endangered [1]. In the first case, ecological functioning and competitively subordinate species (entities of interest) benefit from the impact, while in the second case the native rare species (entity of interest) is harmed by the impact. Thus, the same negative impact on the native prey species can be considered beneficial or harmful in accordance with different values, interests or motivations.  **Native (or indigenous) range:** known or inferred distribution of a species generated from historical (written or verbal) records, or physical evidence of the taxon’s occurrence. Where direct evidence is inadequate to confirm previous occurrence, the existence of suitable habitat within ecologically appropriate proximity to the known range may be taken as adequate evidence of previous occurrence [2]. Note that the definition of native range used here coincides with the definition used by the IUCN to describe the extent of occurrence, i.e. the area contained within the shortest continuous boundary which encompasses all known, inferred and projected sites of present occurrence of a taxon, excluding cases of vagrancy [3,4].  **Population**: group of individuals of the same species that live in a given area and interbreed.   - **Global population**: total number of individuals of a species [5]. - **Sub-population**: geographically or otherwise distinct group of individuals in the global population for which there is little demographic or genetic exchange [5]. - **Local population**: group of individuals within a sub-population. This may encompass all individuals within the sub-population or only some of those individuals. In the latter case, a local population is spatially disjunct from other groups of individuals, but shares individuals with other local populations through natural immigration, in which case it may form part of a metapopulation [5]. - **Local population extinction**: condition that arises from the death of all individuals within a local population. Local population extinction differs from global (species) extinction, which refers to the complete disappearance of a native species from all parts of its range. In situations where a species is only known from one locality, local population extinction may also result in the global species extinction [5,6]. - **Local population re-establishment**: condition that arises from the establishment of a local population within an area belonging to the native range of the species and where it became extinct.   **Taxon**: species or lower taxonomic levels (subspecies, varieties, cultivars, or breeds), including those that are not yet formally described [5].   - **Alien taxon:** taxon moved and introduced either intentionally or unintentionally by human activities beyond the limits of its native range into an area in which it does not naturally occur. This movement allows the species to overcome fundamental biogeographic barriers to its natural dispersal. Common synonyms are exotic, introduced, nonindigenous, or non-native [7]. - **Neonative taxon:** Range-expanding species (i.e. expanding beyond its historic native range) that tracks human-induced environmental change, without specific human assistance [8]. |
| --- |

### **Supporting information B -** **Table reporting contrasting arguments and approaches used to define how alien taxa are considered and should be managed in accordance with different conservation values/motivations.** As multiple values/motivations exist and determine which entities we are interested in (see also Supporting information A), distinct conservation targets can be identified. Note that here we only consider conservation values/motivations that are expressed regardless of any nature’s instrumental (utilitarian) value, i.e. regardless of nature’s contributions to human well-being (see “nature for itself” framing [9]). Also note that such contrasting arguments and approaches are not necessarily mutually exclusive and have been occasionally combined to find a middle-ground to achieve broader conservation goals [10–13].

| Conservation target (or entity of interest) | Conservation approach and key examples from the literature | Values/motivations/rationale behind the conservation approach in relation to alien taxa (as found in key literature) | Consequences for the management of alien taxa | Potential conflicts with other conservation approaches |
| --- | --- | --- | --- | --- |
| Native taxa | Native taxon-based conservation [14–18] (nativism) | “The argument that the threat posed by introduced species is overblown is often buttressed by the observation that native species sometimes also become invasive. An examination of the literature on plant invasions in the United States shows that six times more non-native species have been termed invasive than native species, and that a member of the naturalized non-native pool is 40 times more likely than a native species to be perceived as invasive [...] These results suggest that natives are significantly less likely than non-natives to be problematic for local ecosystems.” [17]  As “alien species have been shown to cause significant changes in native species extinction probabilities, genetic composition of native populations, behaviour patterns, species richness and abundance” [18] native taxa are of primary conservation importance. | Alien taxa that are causing harmful impacts on native taxa should be prioritized for control and eradication. Alien taxa management should be based on blacklisting and “guilty until proven innocent” approaches. | When undertaking conservation efforts to protect native taxa,   - the conservation status (e.g. rarity or endangerment) of alien taxa in their native range might be overlooked. - the ecological function played by alien taxa in the recipient ecosystem might be overlooked. - the right of sentient alien individuals to exist and to not suffer might be overlooked [19]. |
| Native taxa threatened by climate change | Assisted colonization [20–22] | For species that “face significant risk of decline or extinction under climate change […] resource managers and policy-makers must contemplate moving species to sites where they do not currently occur or have not been known to occur in recent history”. For these species, assisted colonization through translocation “may be the only strategy to prevent extinction” [22]. | Alien taxa that are threatened in their native range by climate change should be protected in the alien range. | Assisted colonization might lead to alien populations that themselves become a threat for local native taxa [23,24].  Assisted colonization might lead to alien populations that alter important ecological functions in the recipient ecosystem [23,24].  Assisted colonization might lead to alien populations that cause suffering or deaths to sentient beings in the alien range. |
| Ecosystem functions | Ecosystem functions-based conservation [25–28] | “Some characteristics, for instance nutrient cycling rates, do not necessarily depend on particular species in a particular location and, hence, substitution of one species by another has little impact. There might therefore be no measurable consequence of invasion of a particular species for ecosystem functions” [26]. “Shifting from a species-based to an ecological functions-or socioeconomic-based approach might be an option” [27]. | Both native and alien taxa are included in conservation planning; as long as the main ecosystem functions are maintained in a region, alien taxa can either be protected or controlled. Novel ecosystems can sometimes be accepted. | When undertaking conservation efforts to secure ecosystem functions, the decline of native populations might be acceptable [28].  When undertaking conservation efforts to secure ecosystem functions, the conservation status (e.g. rarity or endangerment) of alien taxa in their native range might be overlooked.  When undertaking conservation efforts to secure ecosystem functions, the right of alien and native sentient beings to exist and to not suffer might be overlooked. |
| Lost ecosystem functions | Rewilding [29–32] | “Rewilding releases outside the indigenous range might be justified if an ecological function has been lost due to extinction, e.g., dispersal of large seeded plants by giant tortoises” [30]. “We here focus on rewilding as trophic rewilding, defined as species introductions to restore top-down trophic interactions and associated trophic cascades to promote self-regulating biodiverse ecosystems” [31]. | Introduction of taxa outside their native range can be pursued to restore lost ecosystem functions. Alien taxa restoring lost ecosystem functions should be accepted. | Rewilding might lead to alien populations that become a threat for local native taxa [33–35].  Rewilding might lead to alien populations that alter important ecosystem functions [33–35].  When undertaking conservation efforts to restore ecosystem functions, the right of alien and native sentient beings to exist and to not suffer might be overlooked. |
| Sentient beings (native or alien) | Compassionate conservation [19,36,37] | “Killing raises pernicious ethical questions regarding the values placed on individuals and populations, suppression of one species to promote another, categorization of species as invasive and inherently malicious, eradication of species from their introduced ranges when their populations are jeopardized in their native ranges, and penalizing others for our own misdeeds […]. Humanity has a moral obligation to help restore threatened populations, but harming sentient beings is a serious matter that cannot be justified solely on the basis of noble aims” [19]. | Sentient alien beings should not be managed in a way that causes their suffering or death. | Not killing or harming sentient alien beings might cause substantial harm to native taxa or lead to their extinction [38–40].  Not killing or harming sentient alien beings might cause alterations to ecosystem functions or lead to their disappearance [38–40].  Not killing or harming native sentient beings might hinder translocation and assisted colonization of predatory taxa, as the latter will inevitably harm native prey [39,40]. |

**Supporting information C -** **Circumstances under which the prevention/mitigation of a decreasing change is considered as a positive change under EICAT+.**

In EICAT+, we also consider as positive impacts (i.e. increasing changes) cases in which an alien species prevents/mitigates decreasing changes, e.g. when the performance of a native individual, the size of a native population, or the occupancy of a native species would have decreased, or decreased to a greater extent, if the alien species had not been introduced. Although some of these positive impacts can be inferred, the prevention of a decreasing change should be assessed under EICAT+ only when there is convincing evidence that a certain biodiversity attribute (e.g. population size) would have decreased, or decreased to a greater extent, in the absence of the alien species. In the case of extinction prevention, for instance, it must be clear that: i) the population was locally heading toward extinction before the introduction of the alien and ii) the alien taxon prevented, through a specific impact mechanism, an extinction that would have occurred in its absence [41,42] (Fig. 2b). Other cases where an alien species may prevent or mitigate decreasing changes are, for instance, those in which the abundance (i.e. a proxy for population size) of a native species declined in the uninvaded (i.e. control) plots but not, or to a lesser extent, in the plots invaded by the alien. Note that positive impacts associated with the prevention/mitigation of a decreasing change will generally be more difficult to study and identify than those associated with actual increasing changes, as the former require extensive data regarding the temporal trend of individual performance, population size or area of occupancy.

**Supporting information D -** **EICAT+ mechanisms and sub-mechanisms by which an alien taxon can cause positive impacts on native biodiversity attributes and examples** **of positive impacts sourced from the literature and assessed under EICAT+** (ML+ = Minimal positive impact, MN+ = Minor positive impact, MO+ = Moderate positive impact, MR+ = Major positive impact, MV+ = Massive positive impact). Rationales behind the formulation of the mechanisms and sub-mechanisms can be found in the main text and in Supporting information G, H and J.

| **EICAT+ mechanisms** | **EICAT+ sub-mechanisms** | **Examples of positive impacts from the literature assessed through EICAT+** |
| --- | --- | --- |
| **1. Provision of trophic resources**  The alien taxon provides trophic resources to native taxa, leading to a positive impact on native taxa. | **1.1. Provision of trophic resources through predation**  The alien taxon provides trophic resources by being preyed upon by native taxa, leading to a positive impact on native taxa. | · The outbreak of an alien moth that represents an abundant trophic subsidy for two native cuckoos did not increase their reproductive success and inter-annual population size [43]. **ML+**  · The physiological condition of a native reef fish improved due to increased prey provided by an abundant alien amphipod [44]. **MN+**  · Over-winter mortality of a near-threatened shorebird decreased due to the introduction of an alien shellfish that acts as a new food source [45]. **MN+**  · Population size of native predatory birds increased after the introduction of an alien crayfish [46] (but see also Ramo et al. [47] who casted doubts on the role of the crayfish as a driver of increase in bird population size). **MO+**  · Population size of three native waterfowl increased after the introduction of alien zebra mussels that were incorporated in the waterfowl diet [48]. **MO+** |
|  | **1.2 Provision of trophic resources through parasitism**  The alien taxon provides trophic resources to a native parasite, leading to a positive impact on native taxa | - |
|  | **1.3 Provision of trophic resources through grazing/herbivory/browsing**  The alien taxon provides trophic resources by being grazed, browsed or consumed through herbivory by native taxa, leading to a positive impact on native taxa. | - |
|  | **1.4 Provision of trophic resources through commensalism/scavenging/detritivory**  The alien taxon provides trophic resources to native taxa through commensalism, scavenging or detritivory, leading to a positive impact on native taxa. | · An alien mussel increased food availability for a native amphipod through biodeposition of faeces and pseudofaeces, thus increasing the amphipod's feeding rate and body length [49]. **MN+** |
|  | **1.5 Provision of trophic resources through mutualism**  The alien taxon provides trophic resources to native taxa through mutualism, leading to a positive impact on native taxa. | · An alien mealybug prolonged worker ant longevity in a native ant by providing honeydew [50]. **MN+**  · An alien forb established a mutualism with native arbuscular mycorrhizal fungi, thus increasing their abundance [51,52]. **MO+**  · Alien shrubs increased the abundance of several native frugivore birds by providing fleshy fruits [53]. **MO+**  · Alien zooxanthellae established a mutualism with bleached native corals, thus preventing the disappearance of photosynthetic corals under stressful thermal conditions due to climate change [54,55]. **MR+** |
| **2. Overcompensation**  The alien taxon causes an overcompensatory response in native taxa, leading to a positive impact on native taxa (see also Supporting information H). | **2.1 Overcompensation to competition**  The alien taxon causes an overcompensatory response to competition in native taxa, leading to a positive impact on native taxa. | - |
|  | **2.2 Overcompensation to predation**  The alien taxon causes an overcompensatory response to predation in native taxa, leading to a positive impact on native taxa. | - |
|  | **2.3 Overcompensation to parasitism**  The alien taxon causes an overcompensatory response to parasitism in native taxa, leading to a positive impact on native taxa. | - |
|  | **2.4 Overcompensation to grazing/herbivory/browsing**  The alien taxon causes an overcompensatory response to grazing/herbivory/browsing in native taxa, leading to a positive impact on native taxa. | - |
| **3. Hybridization**  The alien taxon hybridizes with native taxa without altering their taxonomic status (see also Supporting information J), leading to a positive impact on native taxa. | **3.1 Genetic rescue through hybridization**  The alien taxon hybridizes with native taxa and increases their fitness by introducing new alleles (heterosis), leading to a positive impact on native taxa. | · Hybrids between island and mainland Torrey pine exhibit increased height and fecundity [56]. **MN+**  · The introduction of individuals from a separate subspecies of cougar increased individual survival in a previously declining native population through heterosis [57,58]. **MN+**  · The introduction and hybridization of individuals from a separate subspecies of cougar increased the area of occupancy of a previously declining native population through heterosis [59]. **MV+**  · The Norfolk Island boobook owl, which had dwindled to a single female, was saved from extinction by hybridization with males from a different subspecies of boobook owl [60]. **MV+** |
|  | **3.2 Evolutionary rescue through hybridization**  The alien taxon hybridizes with native taxa and allows them to cope with environmental changes by introducing adaptive genetic variation, leading to a positive impact on native taxa. | · Hybrids between a native plant and a congeneric alien plant were more plastic in rates of photosynthesis and transpiration in response to drought than individuals from the native plant [61]. **MN+**  · Hybrid staghorn corals exhibited increased uptake of algal endosymbionts and photochemical efficiency [62]. **MN+**  · The American chestnut was considered to be “functionally extinct” in its native range, but has been revived by the planting of thousands of resistant hybrids with Chinese chestnut [63]. **MV+** |
| **4. Disease reduction**  The alien taxon reduces incidence in, or transmission, of diseases (caused for instance by pathogens or parasites) to native taxa, leading to a positive impact on the native taxa. | - | · An alien forb increased the growth rate of a native plant likely through the production of allelochemicals suppressing soil borne  pathogenic fungi [64]. **MN+** |
| **5. Dispersal facilitation**  The alien taxon facilitates the dispersal of individuals or propagules (such as seeds, spores, cysts, gametes, pollen) of native taxa, leading to a positive impact on native taxa. | **5.1 Dispersal facilitation through commensalism**  The alien taxon facilitates the dispersal of individuals or propagules (such as seeds, spores, cysts, gametes, pollen) of native taxa through commensalism, leading to a positive impact on native taxa | - |
|  | **5.2 Dispersal facilitation through mutualism**  The alien taxon facilitates the dispersal of individuals or propagules (such as seeds, spores, cysts, gametes, pollen) of native taxa through mutualism, leading to a positive impact on native taxa**.** | · An alien tortoise promoted seedling patches of an endangered tree by facilitating dispersal and germination of seeds through endozoochory [65]. **MN+**  · An alien bird was the sole known pollinator of an endemic native plant [66,67]. **MR+**  · An alien bird dispersed seeds of native plants, replacing the seed dispersal function of native birds that have become extinct [42]. **MR+** |
| **6. Epibiosis or other direct provisioning of habitat**  The accumulation of individuals of native taxa on the surface of the alien taxon, or other direct provisioning of habitat (e.g. provision of refuge, breeding, nesting, foraging, resting, roosting, overwintering sites) lead to positive impacts for native taxa. | **6.1 Epibiosis or other direct provision of habitat through commensalism**  The accumulation of individuals of native taxa on the surface of the alien taxon, or other direct provisions of habitat through commensalism lead to positive impacts for native taxa. | · Reefs created by an alien tubeworm supported a greater biomass of infauna than adjacent sandy areas [68]. **MN+**  · An alien forb increased hunting success and abundance of native spiders by providing foraging habitats [69,70]. **MN+**  · An alien green alga provided a new three-dimensional micro-habitat for a native mussel on breakwaters, thus increasing the abundance of the mussel [71]. **MO+**  · An alien brown alga provided a new substrate for native epiphytic algae, thus increasing their abundance [72]. **MO+**  · An alien bivalve increased the abundance of a native mayfly by providing hard surfaces [73]. **MO+**  · An alien oyster reintroduced lost structural complexity by directly creating biogenic reefs, thus increasing abundance of native reef-  associated sessile suspension feeders [74–76]. **MO+**  · An alien mussel increased the abundance of a native sea slug by providing desiccation refuge [77]. **MO+**  · An alien plant increased the abundance of certain native spiders in dune habitats [78]. **MO+**  · The removal of an alien plant decreased the abundance of a native spider that used the litter produced by the alien plant as a highly  suitable habitat [79]. **MO+**  · An alien tree increased the abundance of multiple native generalist birds, possibly by providing suitable nesting and foraging  habitats [80]. **MO+**  · Alien grasses provided fledging sites to a native bird that was previously extinct in the area [81]. **MR+**  · An alien plant provided refugia for critically endangered, declining and protected native land snails by producing litter that protects  them from predation by rats [41]. **MR+**  · Novel habitats created by alien plants supported distinct insect communities, sometimes including native insect taxa being otherwise  rare or absent [82]. **MR+**  · An alien oyster reintroduced lost structural complexity by directly creating biogenic reefs, thus enhancing colonization of native reef  -associated anthozoans, bryozoans, hydrozoans that are absent in the non-invaded sites [75,76]. **MR+** |
|  | **6.2 Epibiosis or other direct provision of habitat through mutualism**  The accumulation of individuals of native taxa on the surface of the alien taxon, or other direct provisions of habitat through mutualism lead to positive impacts for native taxa. | · An alien seaweed provided foraging habitat, and thus increasing growth, of a native polychaete worm that decorates its tube cap with the alien seaweed. Such decoration behaviour also increased survivorship and growth in the alien seaweed [83]. **MN+** |
| **7. Chemical impact on ecosystem**  The alien taxon causes changes to the chemical characteristics of the native environment (e.g., pH; nutrient and/or water cycling), leading to a positive impact on native taxa. | **-** | · An alien plant increased the biomass of a native grass by increasing the availability of soil nitrogen [84]. **MN+**  · An alien bivalve increased the abundance of a native aquatic plant, likely by modifying oxygen and nitrogen levels through bioturbation [85]. **MO+**  · An alien tree facilitated re-establishment of locally extinct native plants in degraded lands, likely by causing changes in soil chemistry [86]. **MR+**  · An alien green alga modified the quantity, biochemical composition, and nutritional quality of organic detritus in areas characterized by low sediment deposition rates, thus increasing the abundance of native meiofauna [87]. **MO+** |
| **8. Physical impact on ecosystem**  The alien taxon causes changes to the physical characteristics of the native environment (e.g. disturbance or light regimes), leading to a positive impact on native taxa. | **-** | · Alien marine filter feeders generally reduced turbidity and increased light penetration, hence allowing seagrasses and macrophytes to grow at greater depths and thus supporting greater biomasses per unit area by providing more three-dimensional habitat [88]. **MN+**  · An alien tree altered light and temperature regimes (i.e. microclimate), thus increasing canopy cover and facilitating the re-establishment of locally extinct native plants in degraded lands [86]. **MR+** |
| **9. Structural impact on ecosystem**  The alien taxon causes changes to the structural biotope characteristics of the native environment (e.g. changes in architecture or complexity), leading to a positive impact on native taxa. | - | · An alien macroalga created a complex three-dimensional habitat, thus increasing abundance of native epibiotic invertebrates [89]. **MO+**  · Multiple native invertebrates from different feeding guilds (fungivore, herbivore, predator, or saprophage) became more abundant in the ground of bush fragments invaded by three alien plants [90]. **MO+**  · An alien tree facilitated the re-establishment of locally extinct native plants in degraded lands, possibly by causing changes in soil structure [86]. **MR+** |
| **10. Indirect impacts through interactions with other taxa**  The alien taxon interacts with other taxa, native or alien, leading to an indirect positive impact on native taxa. | **10.1 Indirect impacts through interactions with other alien taxa negatively affecting native taxa.**  The alien taxon interacts with other alien taxa that constrain native taxa through negative interactions (e.g. through EICAT mechanisms such as predation, herbivory, competition or toxicity), leading to an indirect positive impact on native taxa. Note that when the alien taxon interacts with other taxa that cause disease in the native taxa (i.e. being pathogens or parasites), the indirect impacts should be classified in accordance with the EICAT+ mechanism 4 (disease reduction). | · An alien canid suppressed the abundance of alien mesopredators, cats and foxes, thus increasing the abundance of native small  mammals and a native owl [91]. **MO+**  · An alien ctenophore predated on another invasive alien ctenophore that had caused the collapse of zooplanktonic communities  and small pelagic fish, thus controlling the invasive population and allowing the recovery of zooplankton and small pelagic fish [92]. **MR+**  ·Alien cats controlled the abundance of alien rabbits, thus preventing substantial damages to native vegetation and local disappearance of native megaherbs and grasses that were indeed observed after the eradication of alien cats [93]. **MR+**  · An alien weevil used as a classical biocontrol agent suppressing an alien invasive aquatic weed, thus allowing the recovery of native aquatic algae and macroinvertebrates [94]. **MR+**  · An alien coccinellid beetle used a as a classical biocontrol agent suppressed and cause the extinction of an alien scale, thus preventing the extinction of a native endemic plant [95,96]. **MV+** |
|  | **10.2 Indirect impacts through interactions with other native taxa negatively affecting native taxa.**  The alien taxon interacts with other native taxa that constrain native taxa through negative interactions (e.g. through EICAT mechanisms such as predation, herbivory, competition or toxicity), leading to an indirect positive impact on native taxa. Note that when the alien taxon interacts with other taxa that cause disease in the native taxa (i.e. being pathogens or parasites), the indirect impacts should be classified in accordance with the EICAT+ mechanism 4 (disease reduction). | · An alien fish decreased the abundance of a competitively dominant native fish, thus increasing the juvenile growth rate of competitively subordinate native fish [97]. **MN+**  · An alien brown alga reduced the abundance of a previously dominant native red alga, thus increasing the abundance of other  native red and brown algae [72]. **MO+**  · An alien ant displaced mutualistic native ants that prune native lianas off from their host plant, thus increasing the abundance of lianas [98]. **MO+**  · A toxic alien toad decreased the abundance of native goannas, thus causing a trophic cascade that increased the population size of four native lizard families preyed upon by goannas [99,100]. **MO+**  · An alien ant increased the abundance of non-predatory native invertebrates, likely by decreasing the abundance of their predators [101]. **MO+**  · An alien ant decreased the abundance of three competitively dominant native ants, thus increasing the abundance of a competitively  subordinate, and locally rare, native ant [102]. **MO+** |
|  | **10.3 Indirect impacts through interactions with other alien taxa positively affecting native taxa.**  The alien taxon interacts with other alien taxa that facilitate native taxa through positive interactions (e.g. through other EICAT+ mechanisms such as provision of trophic resources or dispersal facilitation), leading to an indirect positive impact on native taxa. | **-** |
|  | **10.4 Indirect impacts through interactions with other native taxa positively affecting native taxa.**  The alien taxon interacts with other native taxa that facilitate native taxa through positive interactions (e.g. through other EICAT+ mechanisms such as provision of trophic resources or dispersal facilitation), leading to an indirect positive impact on native taxa. | · An alien shrub increased fruit removal rates of a native plant by increasing the abundance of native frugivores birds [53]. **MN+**  · An alien plant reduced pollen limitation in a native plant by attracting pollinators (magnet species effect), thus increasing the  reproductive success of the native plant [103]. **MN+**  · When compared with a native fruit tree, an alien fruit tree increased below-canopy seed rain and the abundance of seedlings of native plants by attracting a variety of frugivore species [104]. **MN+**  · An alien bivalve increased the abundance of a native bivalve, likely by enhancing, through selective grazing, the preferred diatom  resources (other native taxa) of the native bivalve [85]. **MO+** |

**Supporting information E -** **Table reporting examples sourced from the literature and classified as information that cannot be classified under EICAT+, but that contain information about mechanisms and might set the stage for future studies.** Although these studies described the existence of mechanisms by which alien taxa may cause positive impacts on native taxa, such literature is considered as non-relevant, as it did not measure, or provide information on, biodiversity attributes used in EICAT+ (e.g. performance of individuals or population size). Rationales behind the formulation of the mechanisms and sub-mechanisms can be found in the main text and in Supporting information G, H and J.

| **EICAT+ mechanisms** | **EICAT+ sub-mechanisms** | **Examples of non-relevant information for EICAT+ sourced from the literature** |
| --- | --- | --- |
| **1 Provision of trophic resources**  The alien taxon provides trophic resources to native taxa, leading to a positive impact on native taxa. | **1.1. Provision of trophic resources through predation**  The alien taxon provides trophic resources by being preyed upon by native taxa, leading to a positive impact on native taxa. | · An alien cladoceran constituted an important food source for many native fish [105].  · An invasive bivalve substantially contributed to the diet of many fish and birds [106].  · Native rodents and invertebrates consumed alien toxic toads without showing overt ill effects [107,108].  · Alien gobies were the main prey for native cod and perch, in the diet of multiple piscivorous fish taxa and in the diet of all top predators [109,110].  · An alien goby was a very important food source for a native cormorant, constituting at least 35% of its prey [111].  · An alien apple snail (*Pomacea maculata*) was an important food source for a native ibis*,* constituting ca. 26-40% of its diet [112].  · Alien mammals (European hare/rabbit, wild boar, sheep, and cattle/horse) represented ca. 77, 84 and 99% of the diet of two native fox species and a native couguar [113].  · Alien crayfish was the main food resource for a native otter [114]. |
|  | **1.2 Provision of trophic resources through parasitism**  The alien taxon provides trophic resources by being parasitized by native taxa, leading to a positive impact on native taxa | · Alien phytophagous insects were attacked by complexes of native parasitoids worldwide [115].  · Alien bivalves were adopted as hosts by a native trematode [116].  · In an animal-parasite literature review, native taxa (arthropods, parasitoids, protozoa, and helminths) found to account for 67% of the parasite fauna of alien animal taxa from a range of taxonomic groups [117].  · An alien mink was adopted as host by native nematodes and trematodes [118]. |
|  | **1.3 Provision of trophic resources through grazing/herbivory/browsing**  The alien taxon provides trophic resources by being grazed, browsed or consumed through herbivory by native taxa, leading to a positive impact on native taxa. | · A native shrimp consumed leaves of two alien trees and an alien bamboo in feeding trials [86].  · A salt marsh grass formed extensive monoculture meadows and provided food for many grazers such as geese, ducks and other water birds and wildlife [119].  · Alien plants were fed upon by multiple phytophagous insects [120,121].  · Flowers of an alien plant were consumed by native snail taxa [122].  · An alien seagrass was the preferred prey of a native sea turtle, as revealed by stomach content analysis of stranded and bycaught individuals [123].  · An alien bryozoan was the preferred prey over other native resources by a key native sea urchin, as revealed by stomach content analysis [124]. |
|  | **1.4 Provision of trophic resources through commensalism/scavenging/ detritivory**  The alien taxon provides trophic resources to native taxa through commensalism, scavenging or detritivory, leading to a positive impact on native taxa. | · Alien mammals (sheep/goat, hare/rabbit, red deer and cow/horse) represented 98.5% of the diet of a native scavenging raptor [125].  · Alien toad road-kills were eaten by native scavenging raptors that have learnt to avoid the most toxin-laden body parts of the toad [126].  · Dung of multiple alien taxa were attracted and thought to be used by native dung beetles [127,128].  · Alien parrots made seeds and fruits more available for native fauna due to food wasting [129]. |
|  | **1.5 Provision of trophic resources through mutualism**  The alien taxon provides trophic resources to native taxa through mutualism, leading to a positive impact on native taxa. | · Alien plant species were integrated into existing native plant–pollinator networks worldwide [130–133].  · An alien tree produced seeds consumed and dispersed by native birds, bats, insects, and ants [86].  · Alien plants provided fruit to native birds which have shifted their diet and now frequently forage on the alien plants in an archipelago [42].  · Alien plants produced fleshy fruits that are consumed by native animal taxa worldwide [134]. |
| **2. Overcompensation**  The alien taxon causes an overcompensatory response in native taxa, leading to a positive impact on native taxa (see also Supporting information H). | **2.1 Overcompensation to competition**  The alien taxon causes an overcompensatory response to competition in native taxa, leading to a positive impact on native taxa. | - |
|  | **2.2 Overcompensation to predation**  The alien taxon causes an overcompensatory response to predation in native taxa, leading to a positive impact on native taxa. | - |
|  | **2.3 Overcompensation to parasitism**  The alien taxon causes an overcompensatory response to parasitism in native taxa, leading to a positive impact on native taxa. | - |
|  | **2.4 Overcompensation to grazing/herbivory/browsing**  The alien taxon causes an overcompensatory response to grazing/herbivory/browsing in native taxa, leading to a positive impact on native taxa. | - |
| **3. Hybridization**  The alien taxon hybridizes with native taxa without altering their taxonomic status (see also Supporting information J), leading to a positive impact on native taxa. | **3.1 Genetic rescue through hybridization**  The alien taxon hybridizes with native taxa and increases their fitness by introducing new alleles (heterosis), leading to a positive impact on native taxa. | - |
|  | **3.2 Evolutionary rescue through hybridization**  The alien taxon hybridizes with native taxa and allows them to cope with environmental changes by introducing adaptive genetic variation, leading to a positive impact on native taxa. | - |
| **4. Disease reduction**  The alien taxon reduces incidence in or transmission of diseases (caused for instance by pathogens or parasites) to native taxa, leading to a positive impact on the native taxa. | - | · An alien ant attacked and killed parasites of native trophobiotic coccids, thus reducing the presence of parasites in the native coccids [135].  · An alien vole reduced the presence of the bacteria *Bartonella* in a native wood mouse by dilution effect [136].  · An alien oyster and an alien limpet interfere with the transmission of free-living infective trematode larval stages and thereby mitigated the  parasite burden of native mussels [137].  · Alien toads might have reduced the burden of lungworms in the native fauna by taking up lungworms that otherwise would infect native taxa and that fail to develop in the alien toads [138,139].  · An alien vole reduced the presence of parasitic worms in a native wood mouse by dilution effect [140].  · An alien fish acted as a sink for a parasitic worm of a native fish, thus reducing the parasite burden of the native fish [141]. |
| **5. Dispersal facilitation**  The alien taxon facilitates the dispersal of individuals or propagules (such as seeds, spores, cysts, gametes, pollen) of native taxa, leading to a positive impact on native taxa. | **5.1 Dispersal facilitation through commensalism**  The alien taxon facilitates the dispersal of individuals or propagules (such as seeds, spores, cysts, gametes, pollen) of native taxa through commensalism, leading to a positive impact on native taxa | · Alien bison facilitated dispersal of seeds of native plants by epizoochory [142].  · Livestock ungulates facilitated dispersal of native grassland seeds by epizoochory [143,144]. |
|  | **5.2 Dispersal facilitation through mutualism**  The alien taxon facilitates dispersal of individuals or propagules (such as seeds, spores, cysts, gametes, pollen) of native taxa through mutualism, leading to a positive impact on native taxa**.** | · An alien passerine bird pollinated a native vine formerly pollinated by now extinct and declined bird taxa [145].  · Alien honeybees pollinated an endemic native shrub [146].  · An alien possum facilitated the dispersal and germination of seeds of native plants by feeding on their fruits [147].  · Alien ants dispersed seeds of native plants [148].  · Alien honeybees pollinated multiple native plants that nearly lost all their native pollinators after the introduction of an alien predatory  lizard[149] (which does not prey upon the alien honeybees).  · Alien feral pigs dispersed seeds of a native tree by endozoochory [150].  · Alien pollinators provided pollination service to native plants [133].  Alien parrots dispersed seeds of native plants by mutualistic epizoochory, i.e. by feeding on fruits and seeds that adhered to the surface of parrots after fruit/seed consumption [151]. |
| **6. Epibiosis or other direct provisioning of habitat**  The accumulation of individuals of native taxa on the surface of the alien taxon, or other direct provisioning of habitat (e.g. provision of refuge, breeding, nesting, foraging, resting, roosting, overwintering sites) lead to positive impacts for native taxa. | **6.1 Epibiosis or other direct provision of habitat through commensalism**  The accumulation of individuals of native taxa on the surface of the alien taxon, or other direct provisions of habitat through commensalism lead to positive impacts for native taxa. | · An alien shrub provided breeding sites and shelter to three native birds [152].  · An alien gorse was thought to provide habitat and refugia from predators to native giant weta that were threatened with extinction [153].  · Alien trees provided roosting sites to native monarch butterflies [130].  · Empty shells of an alien bivalve were used by two native hermit crabs [85].  · Alien tree-shrub taxa provided breeding sites to multiple native birds [154]. |
|  | **6.2 Epibiosis or other direct provision of habitat through mutualism**  The accumulation of individuals of native taxa on the surface of the alien taxon, or other direct provisions of habitat through mutualism lead to positive impacts for native taxa. | - |
| **7. Chemical impact on ecosystem**  The alien taxon causes changes to the chemical characteristics of the native environment (e.g., pH; nutrient and/or water cycling), leading to a positive impact on native taxa. | **-** | · An alien burrowing polychaete facilitated the switch from a seasonally hypoxic system back to a normoxic system, alleviating eutrophication, and speeding decomposition of organic matter, thus contributing to the recovery of benthic communities [155].  · An alien brown algae was a suitable adsorbent for Zn(II) and Cd(II) pollutants [156].  · An alien green alga had the capacity to reduce the environmental impact of nitrogen-rich effluents in coastal ecosystems [157]. |
| **8. Physical impact on ecosystem**  The alien taxon causes changes to the physical characteristics of the native environment (e.g. disturbance or light regimes), leading to a positive impact on native taxa. | **-** | · Fossorial activity of alien marmots formed burrows that can be used by small mammals, carnivores, herpetofauna and invertebrates [158,159].  · The appearance of alien zebra mussel is associated with decreases in chlorophyll a and increases in water transparency promoting the expansion of submerged macrophytes [160]. |
| **9. Structural impact on ecosystem**  The alien taxon causes changes to the structural biotope characteristics of the native environment (e.g. changes in architecture or complexity), leading to a positive impact on native taxa. | - | *·*An alien tree increased the rate of primary succession on new lava flows sevenfold, thus disrupting natural plant succession processes and affecting how native and alien taxa colonized the lava flows [161].  *·*An alien parrot built communal nests that were used by native birds and mammals [162]. |
| **10. Indirect impacts through interactions with other taxa**  The alien taxon interacts with other taxa, native or alien, leading to an indirect positive impact on native taxa. | **10.1 Indirect impacts through interactions with other alien taxa negatively affecting native taxa.**  The alien taxon interacts with other alien taxa that constrain native taxa through negative interactions (e.g. through EICAT mechanisms such as predation, herbivory, competition or toxicity), leading to an indirect positive impact on native taxa. Note that when the alien taxon interacts with other taxa that cause disease in the native taxa (i.e. being pathogens or parasites), the indirect impacts should be classified in accordance with the EICAT+ mechanism 4 (disease reduction). | **·** An alien crab fed upon an alien snail, thus acting as a biocontrol agent and facilitating native taxa [163].  · An alien fish fed upon two alien mussels, thus acting as potential biocontrol [164].  · An alien cornetfish has a positive effect on native fish and invertebrates by predating on alien demersal predatory fish, as revealed by a food-web model that considered various indicators such as biomass, ecotrophic efficiency or predation mortality [165]. |
|  | **10.2 Indirect impacts through interactions with other native taxa negatively affecting native taxa.**  The alien taxon interacts with other native taxa that constrain native taxa through negative interactions (e.g. through EICAT mechanisms such as predation, herbivory, competition or toxicity), leading to an indirect positive impact on native taxa. Note that when the alien taxon interacts with other taxa that cause disease in the native taxa (i.e. being pathogens or parasites), the indirect impacts should be classified in accordance with the EICAT+ mechanism 4 (disease reduction). | · An alien ant interacted with herbivorous insects of native plants, thus reducing folivory on the native plants [166].  · In a laboratory setting, a marine alien epizootic limpet fouled native mussels decreasing predation rate by sea stars due to more difficult  handling of fouled mussels in the course of the predation process (i.e. by pulling the two valves apart), thus reducing predation mortality [167].  · An alien ant adopted defensive behaviours to protect a native aphid from native predatory threats [168].  · Alien ants adopted defensive behaviours to protect native trophobionts (e.g. aphids) from predatory threats [148,168].  · An alien parrot participated in a cooperative defence against predators with native birds that used the communal nests built by the parrot [162]. |
|  | **10.3 Indirect impacts through interactions with other alien taxa positively affecting native taxa.**  The alien taxon interacts with other alien taxa that facilitate native taxa through positive interactions (e.g. through other EICAT+ mechanisms such as provision of trophic resources or dispersal facilitation), leading to an indirect positive impact on native taxa. | · An alien rust fungus used as a biocontrol agent against two alien wattles induced the formation of galls that accumulated multi-trophic food webs of native insect taxa [169].  · An alien bud-galling wasp used as a biocontrol agent against two alien wattles induced the formation of galls that accumulated multi-trophic food webs of native insect taxa [169]. |
|  | **10.4 Indirect impacts through interactions with other native taxa positively affecting native taxa.**  The alien taxon interacts with other native taxa that facilitate native taxa through positive interactions (e.g. through other EICAT+ mechanisms such as provision of trophic resources or dispersal facilitation), leading to an indirect positive impact on native taxa. | · Several native plants were visited more frequently by native pollinators (magnet species effect) after the introduction of alien plants [170].  · Alien parrots indirectly facilitated dispersal of native plants by making their seeds and fruits more available for native fauna due to food wasting [129]. |

**Supporting information F - How to attribute a confidence score in EICAT+**

We recommend assigning confidence scores in line with the EICAT guidelines. A confidence score of either low, medium or high is assigned to each individual impact report to reflect the assessor’s judgement that the assigned confidence score is the ‘true’ impact. Under the EICAT guidelines, assessors are instructed to consider five key sources of uncertainty when assigning confidence scores [171,172]. Most importantly, it must be stressed that the confidence scores do not necessarily reflect the quality of an impact observation. For instance, a robust study that finds an effect of an alien species on the performance of a native species (e.g. growth) but does not in any way examine whether this translates to effects to the native species population size cannot be assigned a high confidence score as we have no information as to whether the ‘true’ impact could be higher. Thus, confidence should be assigned in consideration of the question “could the true impact be higher (or lower)?”.

**Supporting information G - Additional information around the rationale behind the formulation of the EICAT+ mechanisms and sub-mechanisms**

In some cases, mechanisms under EICAT+ are identical to those under EICAT; they differ only in that native biodiversity attributes (e.g. individual performance or population size) increase instead of decrease (blue cells in Fig. 3). Examples include *chemical, physical or structural impacts on ecosystems* (e.g. EICAT+ mechanisms 7-8-9) and *hybridization* (mechanism 3, see also Supporting information J for additional details around this mechanism under EICAT+). In other cases, the terminology for the EICAT+ mechanisms has been partially modified in comparison with EICAT, as they differ in whether the alien or native taxon is carrying out the action (yellow cells in Fig. 3). For instance, under EICAT, *predation* describes the consumption of a native prey taxon by an alien taxon. In contrast, under EICAT+, *provision of trophic resources through predation* (sub-mechanism 1.1) might describe the consumption of an alien prey taxon by a native taxon (Fig. 3). Examples of mechanisms unrelated to EICAT mechanisms are those that do not have a negative (-) interaction (e.g. those involving commensalism or mutualism, green cells in Fig.3), e.g. *provision of trophic resources through commensalism/scavenging/detritivory* (sub-mechanism 1.4) or *epibiosis or other direct provisions of habitat through mutualism* (sub-mechanism 6.2). Additionally, we introduce the mechanism *overcompensation*, and its related sub-mechanisms, to cover cases in which native biodiversity attributes (e.g. individual performance or population size) increase through overcompensatory responses towards initially negative interactions (pink cells in Fig. 3, Supporting information H).

**Supporting information H - Additional information about how alien species can cause positive impacts on native biodiversity through overcompensation**

Alien species can cause overcompensatory responses in native taxa at both individual and population levels, thus increasing some biodiversity attributes used in EICAT+, such as individual performance or population size. Overcompensatory responses occurring at individual level have been observed in plant-herbivore interactions, for instance when plants damaged by herbivores have higher fitness when compared with related plants that are undamaged [173,174] (see sub-mechanism 2.4 in Fig. 3). Various degrees of tolerance to consumption have also been detected in host-parasite interactions[175] (see sub-mechanisms 2.3 in Fig. 3). Compensatory and overcompensatory responses at individual level are generally due to allocation trade-offs between contrasting processes such as growth, storage and reproduction [176]. Overcompensatory responses occurring at population level have been observed in age and stage-structured populations characterized by density-dependent traits (e.g. density-dependent mortality) at various stages of the life-cycle [177]. Classical antagonistic interactions such as predation, parasitism and grazing/herbivory/browsing (see sub-mechanisms 2.2, 2.3, 2.4 in Fig. 3) or mutually antagonistic interactions such as competition (see sub-mechanism 2.1 in Fig. 3) can decrease survival at early stages, thus counter-intuitively increasing population size [177,178].

**Supporting information J - Additional information about how alien species can cause positive impacts on native biodiversity through hybridization**

There are two main sub-mechanisms by which hybridization can positively impact native biodiversity: genetic rescue and evolutionary rescue [179]. Genetic rescue refers to an increase in the fitness or size of a native population following the introduction of new alleles through hybridization, whereas in evolutionary rescue, the introgression of adaptive genetic material is required for a population to adapt and survive under a changing environment. Only cases in which the taxonomic status of the impacted species or subspecies has not been lost (i.e. when the impacted taxon can still be taxonomically identified) should be classified as due to genetic or evolutionary rescue [180]. Cases in which the taxonomic status of the native taxon has been lost through hybridization should on the contrary be assessed under EICAT, as these cases indicate negative impacts on native biodiversity [5,171,181]. Both genetic and evolutionary rescue have been experimentally demonstrated in laboratory and greenhouse environments and subsequently confirmed in natural populations [182,183]. Out of the two, genetic rescue has been demonstrated most widely, in part because a single generation of hybridization can enhance the fecundity of an inbred population through heterosis. Evolutionary rescue through hybridization is likely equally common, but it is a slower process and requires several to many generations to document.

Because of the potential risks associated with hybridization between native and alien species, such as genetic or demographic swamping [180], it is important to be able to predict whether hybridization will positively or negatively impact biodiversity. Frankham [183] has provided detailed guidelines for the management of genetic rescue with hybrids and Chan et al. [179] offers a decision tree that can apply to both genetic and evolutionary rescue. Both papers make the point that small and isolated populations that lack sufficient genetic variation for survival and/or adaptation to anticipated environmental change are likely to benefit from hybridization, especially if the alien donor is closely related and genetically diverse.

**Supporting information K - References used in the Supporting information**

1. Vimercati G, Kumschick S, Probert A, Volery L, Bacher S. The importance of assessing positive and beneficial impacts of alien species. NeoBiota. 2020;65: 525–545. doi: 10.3897/neobiota.62.52793

2. IUCN/SSC. Guidelines for Reintroductions and Other Conservation Translocations. Version 1.0. Gland, Switzerland: IUCN Species Survival Commission; 2013.

3. IUCN. IUCN Red List Categories and Criteria: Version 3.1. Second. International Union for the Conservation of Nature SSC, editor. Gland, Switzerland and Cambridge, UK: IUCN: IUCN; 2012. Available: https://portals.iucn.org/library/node/10315

4. IUCN. Glossary of definitions. In: IUCN Definitions ENGLISH [Internet]. 2021 [cited 17 Aug 2021]. doi:10.1017/cbo9780511558207.034

5. IUCN. IUCN EICAT Categories and Criteria. The Environmental Impact Classification for Alien Taxa. IUCN EICAT Categories and Criteria: first edition. 2020. doi.org/10.2305/IUCN.CH.2020.05.en

6. Begon M, Townsend CR, Harper JL. Ecology: From individuals to ecosystems. Four. Wiley-Blackwell; 2005.

7. Blackburn TM, Pyšek P, Bacher S, Carlton JT, Duncan RP, Jarošík V, et al. A proposed unified framework for biological invasions. Trends Ecol Evol. 2011;26: 333–339. doi:10.1016/j.tree.2011.03.023

8. Essl F, Dullinger S, Genovesi P, Hulme PE, Jeschke JM, Katsanevakis S, et al. A Conceptual Framework for Range-Expanding Species that Track Human-Induced Environmental Change. Bioscience. 2019;69: 908–919. doi:10.1093/biosci/biz101

9. Mace GM. Whose conservation? Changes in the perception and goals of nature conservation require a solid scientific basis. Science. 2014;245: 1558–1560. doi:10.1126/science.1254704

10. Seddon PJ. From Reintroduction to Assisted Colonization: Moving along the Conservation Translocation Spectrum. Restor Ecol. 2010;18: 796–802. doi:10.1111/j.1526-100X.2010.00724.x

11. Loss SR, Terwilliger LA, Peterson AC. Assisted colonization: Integrating conservation strategies in the face of climate change. Biol Conserv. 2011;144: 92–100. doi:10.1016/j.biocon.2010.11.016

12. Lawler JJ, Olden JD. Reframing the debate over assisted colonization. Front Ecol Environ. 2011;9: 569–574. doi:10.1890/100106

13. Shackelford N, Hobbs RJ, Heller NE, Hallett LM, Seastedt TR. Finding a middle-ground: The native/non-native debate. Biol Conserv. 2013;158: 55–62. doi:10.1016/j.biocon.2012.08.020

14. Ruesink JL, Parker IM, Groom MJ, Kareiva PM. Reducing the risks of nonindigenous species introductions. Guilty until proven innocent. Bioscience. 1995;45: 465–477. doi:10.2307/1312790

15. Simberloff D. Non-native species DO threaten the natural environment! J Agric Environ Ethics. 2005;18: 595–607. doi:10.1007/s10806-005-2851-0

16. Simberloff D. Non-natives: 141 scientists object. Nature. 2011.

17. Simberloff D, Souza L, Nũnez MA, Barrios-Garcia MN, Bunn W. The natives are restless, but not often and mostly when disturbed. Ecology. 2012;93: 598–607. doi:10.1890/11-1232.1

18. Blackburn TM, Essl F, Evans T, Hulme PE, Jeschke JM, Kühn I, et al. A Unified Classification of Alien Species Based on the Magnitude of their Environmental Impacts. PLoS Biol. 2014;12. doi:10.1371/journal.pbio.1001850

19. Wallach AD, Bekoff M, Nelson MP, Ramp D. Promoting predators and compassionate conservation. Conserv Biol. 2015;29: 1481–1484. doi:10.1111/cobi.12525

20. McLachlan JS, Hellmann JJ, Schwartz MW. A framework for debate of assisted migration in an era of climate change. Conserv Biol. 2007;21: 297–302. doi:10.1111/j.1523-1739.2007.00676.x

21. Hunter ML. Climate change and moving species: Furthering the debate on assisted colonization. Conserv Biol. 2007;21: 1356–1358. doi:10.1111/j.1523-1739.2007.00780.x

22. Hoegh-Guldberg O, Hughes L, McIntyre S, Lindenmayer DB, Parmesan C, Possingham HP, et al. Assisted colonization and rapid climate change. Science. 2008;321: 345–346. doi:10.1126/science.1157897

23. Ricciardi A, Simberloff D. Assisted colonization is not a viable conservation strategy. Trends Ecol Evol. 2009;24: 248–253. doi:10.1016/j.tree.2008.12.006

24. Sandler R. The Value of Species and the Ethical Foundations of Assisted Colonization. Conserv Biol. 2010;24: 424–431. doi:10.1111/j.1523-1739.2009.01351.x

25. Hobbs RJ, Cramer VA. Restoration Ecology: Interventionist Approaches for Restoring and Maintaining Ecosystem Function in the Face of Rapid Environmental Change. Annu Rev Environ Resour. 2008;33: 39–61. doi:10.1146/annurev.environ.33.020107.113631

26. Hobbs RJ, Higgs E, Harris JA. Novel ecosystems: implications for conservation and restoration. Trends Ecol Evol. 2009;24: 599–605. doi:10.1016/j.tree.2009.05.012

27. Mačic V, Albano PG, Almpanidou V, Claudet J, Corrales X, Essl F, et al. Biological invasions in conservation planning: A global systematic review. Front Mar Sci. 2018;5. doi:10.3389/fmars.2018.00178

28. Rilov G, Fraschetti S, Gissi E, Pipitone C, Badalamenti F, Tamburello L, et al. A fast-moving target: achieving marine conservation goals under shifting climate and policies. Ecol Appl. 2020;30: 1–14. doi:10.1002/eap.2009

29. Donlan J. Re-wilding North America. Nature. 2005;436: 913–914. doi:10.1038/436913a

30. Seddon PJ. Griffiths CJ, Soorae PS, Armstrong DP. Reversing defaunation: Restoring species in a changing world. Science. 2014; 345: 406-412. doi:10.1126/science.1251818

31. Svenning JC, Pedersen PBM, Donlan CJ, Ejrnæs R, Faurby S, Galetti M, et al. Science for a wilder Anthropocene: Synthesis and future directions for trophic rewilding research. Proc Natl Acad Sci U S A. 2016;113: 898–906. doi:10.1073/pnas.1502556112

32. Perino A, Pereira HM, Navarro LM, Fernández N, Bullock JM, Ceauşu S, et al. Rewilding complex ecosystems. Science. 2019; 364: doi:10.1126/science.aav5570

33. Rubenstein DR, Rubenstein DI, Sherman PW, Gavin TA. Pleistocene Park: Does re-wilding North America represent sound conservation for the 21st century? Biol Conserv. 2006;132: 232–238. doi:10.1016/j.biocon.2006.04.003

34. Nogués-Bravo D, Simberloff D, Rahbek C, Sanders NJ. Rewilding is the new pandora’s box in conservation. Curr Biol. 2016;26: R87–R91. doi:10.1016/j.cub.2015.12.044

35. Derham TT, Duncan RP, Johnson CN, Jones ME. Hope and caution: Rewilding to mitigate the impacts of biological invasions. Philosophical Transactions of the Royal Society B: Biological Sciences. 2018. doi:10.1098/rstb.2018.0127

36. Ramp D, Bekoff M. Compassion as a practical and evolved ethic for conservation. Bioscience. 2015;65: 323–327. doi:10.1093/biosci/biu223

37. Wallach AD, Bekoff M, Batavia C, Nelson MP, Ramp D. Summoning compassion to address the challenges of conservation. Conserv Biol. 2018;32: 1255–1265. doi:10.1111/cobi.13126

38. Driscoll DA, Watson MJ. Science denialism and compassionate conservation: response to Wallach et al. 2018. Conserv Biol. 2019;33: 777–780. doi:10.1111/cobi.13273

39. Hayward MW, Callen A, Allen BL, Ballard G, Broekhuis F, Bugir C, et al. Deconstructing compassionate conservation. Conserv Biol. 2019;33: 760–768. doi:10.1111/cobi.13366

40. Callen A, Hayward MW, Klop-Toker K, Allen BL, Ballard G, Broekhuis F, et al. Envisioning the future with ‘compassionate conservation’: An ominous projection for native wildlife and biodiversity. Biol Conserv. 2020;241: 108365. doi:10.1016/j.biocon.2019.108365

41. Chiba S. Invasive non-native species’ Provision of refugia for endangered native species. Conserv Biol. 2010;24: 1141–1147. doi:10.1111/j.1523-1739.2010.01457.x

42. Kawakami K, Mizusawa L, Higuchi H. Re-established mutualism in a seed-dispersal system consisting of native and introduced birds and plants on the Bonin Islands, Japan. Ecol Res. 2009;24: 741–748. doi:10.1007/s11284-008-0543-8

43. Barber N, Marquis R, Tori W. Invasive prey impacts the abundance and distribution of native predators. Ecology. 2008;89: 2678–2683.

44. Page HM, Dugan JE, Schroeder DM, Nishimoto MM, Love MS, Hoesterey JC. Trophic links and condition of a temperate reef fish: Comparisons among offshore oil platform and natural reef habitats. Mar Ecol Prog Ser. 2007;344: 245–256. doi:10.3354/meps06929

45. Caldow RWG, Stillman RA, Dit Durell SEAL V., West AD, McGrorty S, Goss-Custard JD, et al. Benefits to shorebirds from invasion of a non-native shellfish. Proc R Soc B Biol Sci. 2007;274: 1449–1455. doi:10.1098/rspb.2007.0072

46. Tablado Z, Tella JL, Sánchez-Zapata JA, Hiraldo F. The paradox of the Long-Term positive effects of a north american crayfish on a european community of predators. Conserv Biol. 2010;24: 1230–1238. doi:10.1111/j.1523-1739.2010.01483.x

47. Ramo C, Aguilera E, Figuerola J, Má̃nez M, Green AJ. Long-term population trends of colonial wading birds breeding in doñana (Sw Spain) in relation to environmental and anthropogenic factors. Ardeola. 2013;60: 305–326. doi:10.13157/arla.60.2.2013.305

48. Petrie SA, Knapton RW. Rapid Increase and Subsequent Decline of Zebra and Quagga Mussels in Long Point Bay, Lake Erie: Possible Influence of Waterfowl Predation Scott. J Great Lakes Res. 1999;25: 772–782. Available: http://longpointwaterfowl.org/wp-content/uploads/2011/05/Petrie-Knapton-1999-Mussels-Waterfowl-Predation.pdf

49. Gergs R, Rothhaupt KO. Feeding rates, assimilation efficiencies and growth of two amphipod species on biodeposited material from zebra mussels. Freshw Biol. 2008;53: 2494–2503. doi:10.1111/j.1365-2427.2008.02077.x

50. Feng DD, Michaud JP, Li P, Zhou ZS, Xu ZF. The native ant, *Tapinoma melanocephalum*, improves the survival of an invasive mealybug, *Phenacoccus solenopsis*, by defending it from parasitoids. Sci Rep. 2015;5: 1–8. doi:10.1038/srep15691

51. Jin L, Gu Y, Xiao M, Chen J, Li B. The history of *Solidago canadensis* invasion and the development of its mycorrhizal associations in newly-reclaimed land. Funct Plant Biol. 2004;31: 979–986. doi:10.1071/FP04061

52. Zhang Q, Yang R, Tang J, Yang H, Hu S, Chen X. Positive feedback between mycorrhizal fungi and plants influences plant invasion success and resistance to invasion. PLoS One. 2010;5. doi:10.1371/journal.pone.0012380

53. Gleditsch JM, Carlo TA. Fruit quantity of invasive shrubs predicts the abundance of common native avian frugivores in central Pennsylvania. Divers Distrib. 2011;17: 244–253. doi:10.1111/j.1472-4642.2010.00733.x

54. LaJeunesse TC, Smith RT, Finney J, Oxenford H. Outbreak and persistence of opportunistic symbiotic dinoflagellates during the 2005 Caribbean mass coral “bleaching” event. Proc R Soc B Biol Sci. 2009;276: 4139–4148. doi:10.1098/rspb.2009.1405

55. Pettay D, Wham D, Smith RT, Iglesias-Prieto R, LaJeunesse TC. Microbial invasion of the Caribbean by an Indo-Pacific coral zooxanthella. Proc Natl Acad Sci U S A. 2015;112: 7513–7518. doi:10.1073/pnas.1502283112

56. Hamilton JA, Royauté R, Wright JW, Hodgskiss P, Ledig FT. Genetic conservation and management of the California endemic, Torrey pine (*Pinus torreyana* Parry): Implications of genetic rescue in a genetically depauperate species. Ecol Evol. 2017;7: 7370–7381. doi:10.1002/ece3.3306

57. Hostetler JA, Onorato DP, Nichols JD, Johnson WE, Roelke ME, O’Brien SJ, et al. Genetic introgression and the survival of Florida panther kittens. Biol Conserv. 2010;143: 2789–2796. doi:10.1016/j.biocon.2010.07.028

58. Benson JF, Hostetler JA, Onorato DP, Johnson WE, Roelke ME, O’Brien SJ, et al. Intentional genetic introgression influences survival of adults and subadults in a small, inbred felid population. J Anim Ecol. 2011;80: 958–967. doi:10.1111/j.1365-2656.2011.01809.x

59. Pimm SL, Dollar L, Bass OL. The genetic rescue of the Florida panther. Anim Conserv. 2006;9: 115–122. doi:10.1111/j.1469-1795.2005.00010.x

60. Garnett ST, Olsen P, Butchart SHM, Hoffmann AA. Did hybridization save the Norfolk Island boobook owl *Ninox novaeseelandiae undulata*? Oryx. 2011;45: 500–504. doi:10.1017/S0030605311000871

61. Brock MT, Galen C. Drought tolerance in the alpine dandelion, *Taraxacum ceratophorum* (Asteraceae), its exotic congener *T. officinale*, and interspecific hybrids under natural and experimental conditions. Am J Bot. 2005;92: 1311–1321. doi:10.3732/ajb.92.8.1311

62. Chan WY, Peplow LM, Menéndez P, Hoffmann AA, van Oppen MJH. Interspecific hybridization may provide novel opportunities for coral reef restoration. Front Mar Sci. 2018;5: 1–15. doi:10.3389/fmars.2018.00160

63. Clark SL, Schlarbaum SE, Saxton AM, Hebard F V. Establishment of American chestnuts (*Castanea dentata*) bred for blight (*Cryphonectria parasitica*) resistance: influence of breeding and nursery grading. New For. 2016;47: 243–270. doi:10.1007/s11056-015-9512-6

64. Schittko C, Wurst S. Above- and belowground effects of plant-soil feedback from exotic *Solidago canadensis* on native *Tanacetum vulgare*. Biol Invasions. 2014;16: 1465–1479. doi:10.1007/s10530-013-0584-y

65. Griffiths CJ, Hansen DM, Jones CG, Zuël N, Harris S. Resurrecting extinct interactions with extant substitutes. Curr Biol. 2011;21: 762–765. doi:10.1016/j.cub.2011.03.042

66. Olesen JM, Ronsted N, Tolderlund U, Cornett C, Molgaard P, Madsen J, et al. Mauritian red nectar remains a mystery [3]. Nature. 1998;393: 529. doi:10.1038/31128

67. Olesen JM, Eskildsen LI, Venkatasamy S. Invasion of pollination networks on oceanic islands: Importance of invader complexes and endemic super generalists. Divers Distrib. 2002;8: 181–192. doi:10.1046/j.1472-4642.2002.00148.x

68. McQuaid KA, Griffiths CL. Alien reef-building polychaete drives long-term changes in invertebrate biomass and diversity in a small, urban estuary. Estuar Coast Shelf Sci. 2014;138: 101–106. doi:10.1016/j.ecss.2013.12.016

69. Dudek K, Michlewicz M, Dudek M, Tryjanowski P. Invasive Canadian goldenrod (*Solidago canadensis* L.) as a preferred foraging habitat for spiders. Arthropod Plant Interact. 2016;10: 377–381. doi:10.1007/s11829-016-9455-7

70. Bauer T, Bäte DA, Kempfer F, Schirmel J. Differing impacts of two major plant invaders on urban plant-dwelling spiders (Araneae) during flowering season. Biol Invasions. 2021;0123456789. doi:10.1007/s10530-020-02452-w

71. Bulleri F, Airoldi L, Branca GM, Abbiati M. Positive effects of the introduced green alga, *Codium fragile* ssp. *tomentosoides*, on recruitment and survival of mussels. Mar Biol. 2006;148: 1213–1220. doi:10.1007/s00227-005-0181-4

72. Sánchez Í, Fernández C, Arrontes J. Long-term changes in the structure of intertidal assemblages after invasion by *Sargassum muticum* (Phaeophyta). J Phycol. 2005;41: 942–949. doi:10.1111/j.1529-8817.2005.00122.x

73. Werner S, Rothhaupt KO. Effects of the invasive bivalve *Corbicula fluminea* on settling juveniles and other benthic taxa. J North Am Benthol Soc. 2007;26: 673–680. doi:10.1899/07-017R.1

74. Kochmann J, Buschbaum C, Volkenborn N, Reise K. Shift from native mussels to alien oysters: Differential effects of ecosystem engineers. J Exp Mar Bio Ecol. 2008;364: 1–10. doi:10.1016/j.jembe.2008.05.015

75. Markert A, Wehrmann A, Kröncke I. Recently established *Crassostrea*-reefs versus native *Mytilus*-beds: Differences in ecosystem engineering affects the macrofaunal communities (Wadden Sea of Lower Saxony, southern German Bight). Biol Invasions. 2010;12: 15–32. doi:10.1007/s10530-009-9425-4

76. Troost K. Causes and effects of a highly successful marine invasion: Case-study of the introduced Pacific oyster *Crassostrea gigas* in continental NW European estuaries. J Sea Res. 2010;64: 145–165. doi:10.1016/j.seares.2010.02.004

77. Sadchatheeswaran S, Branch GM, Robinson TB. Changes in habitat complexity resulting from sequential invasions of a rocky shore: implications for community structure. Biol Invasions. 2015;17: 1799–1816. doi:10.1007/s10530-014-0837-4

78. Gomes M, Carvalho JC, Gomes P. Invasive plants induce the taxonomic and functional replacement of dune spiders. Biol Invasions. 2018;20: 533–545. doi:10.1007/s10530-017-1555-5

79. Braschi J, Hélard O, Mazzia C, Oger P, Ponel P, Buisson E. Impacts of the removal of invasive *Carpobrotus* on spider assemblage dynamics. Biodivers Conserv. 2021;30: 497–518. doi:10.1007/s10531-020-02102-6

80. Hanzelka J, Reif J. Responses to the black locust (*Robinia pseudoacacia*) invasion differ between habitat specialists and generalists in central European forest birds. J Ornithol. 2015;156: 1015–1024. doi:10.1007/s10336-015-1231-4

81. Jones ZF, Bock CE. The Botteri’s Sparrow and exotic Arizona grasslands: An ecological trap or habitat regained? Condor. 2005;107: 731–741. doi:10.1650/7741.1

82. Padovani RJ, Salisbury A, Bostock H, Roy DB, Thomas CD. Introduced plants as novel Anthropocene habitats for insects. Glob Chang Biol. 2020;26: 971–988. doi:10.1111/gcb.14915

83. Kollars NM, Byers JE, Sotka EE. Invasive décor: An association between a native decorator worm and a non-native seaweed can be mutualistic. Mar Ecol Prog Ser. 2016;545: 135–145. doi:10.3354/meps11602

84. Quinos PM, Insausti P, Soriano A. Facilitative effect of Lotus tenuis on Paspalum dilatatum in a lowland grassland of Argentina. Oecologia. 1998;114: 427–431. doi:10.1007/s004420050466

85. Wonham MJ, O’Connor M, Harley CDG. Positive effects of a dominant invader on introduced and native mudflat species. Mar Ecol Prog Ser. 2005;289: 109–116. doi:10.3354/meps289109

86. Lugo AE. The outcome of alien tree invasions in Puerto Rico. Front Ecol Environ. 2004;2: 265–273. doi:10.1890/1540-9295(2004)002[0265:TOOATI]2.0.CO;2

87. Rizzo L, Pusceddu A, Bianchelli S, Fraschetti S. Potentially combined effect of the invasive seaweed *Caulerpa cylindracea* (Sonder) and sediment deposition rates on organic matter and meiofaunal assemblages. Mar Environ Res. 2020;159: 104966. doi:10.1016/j.marenvres.2020.104966

88. Katsanevakis S, Wallentinus I, Zenetos A, Leppäkoski E, Çinar ME, Oztürk B, et al. Impacts of invasive alien marine species on ecosystem services and biodiversity: A pan-European review. Aquat Invasions. 2014;9: 391–423. doi:10.3391/ai.2014.9.4.01

89. Nohrén E, Odelgård E. Response of epibenthic faunal assemblages to varying vegetation structures and habitat patch size. Aquat Biol. 2010;9: 139–148. doi:10.3354/ab00247

90. Bassett IE. Impacts on invertebrate fungivores: A predictable consequence of ground-cover weed invasion? Biodivers Conserv. 2014;23: 791–810. doi:10.1007/s10531-014-0634-5

91. Rees JD, Rees GL, Kingsford RT, Letnic M. Indirect commensalism between an introduced apex predator and a native avian predator. Biodivers Conserv. 2019;28: 2687–2700. doi:10.1007/s10531-019-01787-8

92. Shiganova TA, Bulgakova Y V., Volovik SP, Mirzoyan ZA, Dudkin SI. The new invader *Beroe ovata* Mayer 1912 and its effect on the ecosystem in the northeastern Black Sea. Hydrobiologia. 2001;451: 187–197. doi:10.1023/A:1011823903518

93. Bergstrom DM, Lucieer A, Kiefer K, Wasley J, Belbin L, Pedersen TK, et al. Indirect effects of invasive species removal devastate World Heritage Island. J Appl Ecol. 2009;46: 73–81. doi:10.1111/j.1365-2664.2008.01601.x

94. Motitsoe SN, Coetzee JA, Hill JM, Hill MP. Biological control of *Salvinia molesta* (D.S. Mitchell) drives aquatic ecosystem recovery. Diversity. 2020;12. doi:10.3390/D12050204

95. Cronk QCB. Extinction and survival in the endemic vascular flora of ascension island. Biol Conserv. 1980;17: 207–219. doi:10.1016/0006-3207(80)90056-7

96. Fowler S V. Biological control of an exotic scale, *Orthezia insignis* Browne (Homoptera: Ortheziidae), saves the endemic gumwood tree, *Commidendrum robustum* (Roxb.) DC. (Asteraceae) on the island of St. Helena. Biol Control. 2004;29: 367–374. doi:10.1016/j.biocontrol.2003.06.002

97. Kindinger TL. Invasive predator tips the balance of symmetrical competition between native coral-reef fishes. Ecology. 2018;99: 792–800. doi:10.1002/ecy.2173

98. Mikissa JB, Jeffery K, Fresneau D, Mercier JL. Impact of an invasive alien ant, *Wasmannia auropunctata* Roger., on a specialised plant-ant mutualism, *Barteria fistulosa* Mast. and *Tetraponera aethiops* F. Smith., in a Gabon forest. Ecol Entomol. 2013;38: 580–584. doi:10.1111/een.12057

99. Feit B, Gordon CE, Webb JK, Jessop TS, Laffan SW, Dempster T, et al. Invasive cane toads might initiate cascades of direct and indirect effects in a terrestrial ecosystem. Biol Invasions. 2018;20: 1833–1847. doi:10.1007/s10530-018-1665-8

100. Feit B, Dempster T, Jessop TS, Webb JK, Letnic M. A trophic cascade initiated by an invasive vertebrate alters the structure of native reptile communities. Glob Chang Biol. 2020;26: 2829–2840. doi:10.1111/gcb.15032

101. Goodman M, Warren RJ. Non-native ant invader displaces native ants but facilitates non-predatory invertebrates. Biol Invasions. 2019;21: 2713–2722. doi:10.1007/s10530-019-02005-w

102. Palmer TM, Riginos C, Milligan PD, Hays BR, Pietrek AG, Maiyo NJ, et al. Frenemy at the gate: Invasion by *Pheidole megacephala* facilitates a competitively subordinate plant ant in Kenya. Ecology. 2021;102: 1–13. doi:10.1002/ecy.3230

103. da Silva EM, King VM, Russell-Mercier JL, Sargent RD. Evidence for pollen limitation of a native plant in invaded communities. Oecologia. 2013;172: 469–476. doi:10.1007/s00442-012-2513-7

104. Gomes M, Cazetta E, Bovendorp R, Faria D. Jackfruit trees as seed attractors and nurses of early recruitment of native plant species in a secondary forest in Brazil. Plant Ecol. 2021;222: 1143–1155. doi:10.1007/s11258-021-01167-9

105. Kotta J, Kotta I, Simm M, Lankov A, Lauringson V, Põllumäe A, et al. Ecological consequences of biological invasions: Three invertebrate case studies in the north-eastern Baltic Sea. Helgol Mar Res. 2006;60: 106–112. doi:10.1007/s10152-006-0027-6

106. Tulp I, Craeymeersch J, Leopold M, van Damme C, Fey F, Verdaat H. The role of the invasive bivalve *Ensis directus* as food source for fish and birds in the Dutch coastal zone. Estuar Coast Shelf Sci. 2010;90: 116–128. doi:10.1016/j.ecss.2010.07.008

107. Cabrera-Guzmán E, Crossland MR, Shine R. Invasive Cane Toads as Prey for Native Arthropod Predators in Tropical Australia. Herpetol Monogr. 2015;29: 28–39. doi:10.1655/HERPMONOGRAPHS-D-13-00007

108. Cabrera-Guzmán E, Crossland MR, Pearson D, Webb JK, Shine R. Predation on invasive cane toads (*Rhinella marina*) by native Australian rodents. J Pest Sci (2004). 2015;88: 143–153. doi:10.1007/s10340-014-0586-2

109. Liversage K, Nurkse K, Kotta J, Järv L. Environmental heterogeneity associated with European perch (*Perca fluviatilis*) predation on invasive round goby (*Neogobius melanostomus*). Mar Environ Res. 2017;132: 132–139. doi:10.1016/j.marenvres.2017.10.017

110. Behrens JW, van Deurs M, Puntila-Dodd R, Florin A-B. Round goby – a threat or a new resource? 2019. doi:10.6027/no2019-037

111. Oesterwind D, Bock C, Förster A, Gabel M, Henseler C, Kotterba P, et al. Predator and prey: the role of the round goby *Neogobius melanostomus* in the western Baltic. Mar Biol Res. 2017;13: 188–197. doi:10.1080/17451000.2016.1241412

112. Bertolero A, Navarro J. A native bird as a predator for the invasive apple snail, a novel rice field invader in Europe. Aquat Conserv Mar Freshw Ecosyst. 2018;28: 1099–1104. doi:10.1002/aqc.2917

113. Novaro AJ, Funes MC, Walker RS. Ecological extinction of native prey of a carnivore assemblage in Argentina Patagonia. Biol Conserv. 2000;92: 25–33. Available: www.elsevier.com/locate/biocon

114. Delibes M, Adrián I. Effects of crayfish introduction on Otter *Lutra lutra* food in the Doñana National Park, SW Spain. Biol Conserv. 1987;42: 153–159. doi:10.1016/0006-3207(87)90021-8

115. Cornell H V., Hawkins BA. Accumulation of native parasitoid species on introduced herbivores: a comparison of hosts as natives and hosts as invaders. Am Nat. 1993;141: 847–865. doi:10.1086/285512

116. Krakau M, Thieltges DW, Reise K. Native parasites adopt introduced bivalves of the North Sea. Biol Invasions. 2006;8: 919–925. doi:10.1007/s10530-005-4734-8

117. Kelly DW, Paterson RA, Townsend CR, Poulin R, To DM, 1. Parasite spillback: A neglected concept in invasion ecology? Ecology. 2009;90: 2047–2056. Available: http://izt.ciens.ucv.ve/ecologia/Archivos/ECOLOGIA_DE _POBLACIONES_Hasta 2004/ECO_POB 2007/ECOPO7_2007/Carey et al 2007.pdf

118. Kołodziej-Sobocińska M, Brzeziński M, Niemczynowicz A, Zalewski A. High parasite infection level in non-native invasive species: it is just a matter of time. Ecography (Cop). 2018;41: 1283–1294. doi:10.1111/ecog.03362

119. Nehring S, Andersen H. Invasive Alien Species Fact Sheet - *Spartina anglica*. Nobanis. 2006; 1–13. Available: www.nobanis.org

120. Memmott J, Fowler S V., Paynter Q, Sheppard AW, Syrett P. The invertebrate fauna on broom, *Cytisus scoparius*, in two, native and two exotic habitats. Acta Oecologica. 2000;21: 213–222. doi:10.1016/S1146-609X(00)00124-7

121. Heiselmeyer T, Boulton A, Beauchamp V. The Relationship between Native Insects and an Invasive Grass (*Oplismenus undulatifolius*) in the Mid-Atlantic United States. Northeast Nat. 2019;26: 183–201. doi:10.1656/045.026.0116

122. Rodríguez J, Cordero-Rivera A, González L. Impacts of the invasive plant *Carpobrotus edulis* on herbivore communities on the Iberian Peninsula. Biol Invasions. 2021;23: 1425–1441. doi:10.1007/s10530-020-02449-5

123. Palmer JL, Beton D, Çiçek BA, Davey S, Duncan EM, Fuller WJ, et al. Dietary analysis of two sympatric marine turtle species in the eastern Mediterranean. Mar Biol. 2021;168: 1–16. doi:10.1007/s00227-021-03895-y

124. Camps-Castellà J, Romero J, Prado P. Trophic plasticity in the sea urchin *Paracentrotus lividus*, as a function of resource availability and habitat features. Mar Ecol Prog Ser. 2020;637: 71–85. doi:10.3354/meps13235

125. Lambertucci SA, Trejo A, Di Martino S, Sánchez-zapata JA, Donázar JA, Hiraldo F. Spatial and temporal patterns in the diet of the Andean condor: Ecological replacement of native fauna by exotic species. Anim Conserv. 2009;12: 338–345. doi:10.1111/j.1469-1795.2009.00258.x

126. Beckmann C, Shine R. Toad’s tongue for breakfast: Exploitation of a novel prey type, the invasive cane toad, by scavenging raptors in tropical Australia. Biol Invasions. 2011;13: 1447–1455. doi:10.1007/s10530-010-9903-8

127. Jones AG, Forgie SA, Scott DJ, Beggs JR. Generalist dung attraction response in a New Zealand dung beetle that evolved with an absence of mammalian herbivores. Ecol Entomol. 2012;37: 124–133. doi:10.1111/j.1365-2311.2012.01344.x

128. Stavert J, Drayton B, Beggs J, Gaskett A. The volatile organic compounds of introduced and native dung and carrion and their role in dung beetle foraging behaviour. Ecol Entomol. 2014;39: 556–565. doi:10.1111/een.12133

129. Sebastián-González E, Hiraldo F, Blanco G, Hernández-Brito D, Romero-Vidal P, Carrete M, et al. The extent, frequency and ecological functions of food wasting by parrots. Sci Rep. 2019;9: 1–11. doi:10.1038/s41598-019-51430-3

130. Graves SD, Shapiro AM. Exotics as host plants of the California butterfly fauna. Biol Conserv. 2003;110: 413–433. doi:10.1016/S0006-3207(02)00233-1

131. Vilà M, Bartomeus I, Dietzsch AC, Petanidou T, Steffan-Dewenter I, Stout JC, et al. Invasive plant integration into native plant-pollinator networks across Europe. Proc R Soc B Biol Sci. 2009;276: 3887–3893. doi:10.1098/rspb.2009.1076

132. Drossart M, Michez D, Vanderplanck M. Invasive plants as potential food resource for native pollinators: A case study with two invasive species and a generalist bumble bee. Sci Rep. 2017;7: 1–12. doi:10.1038/s41598-017-16054-5

133. Traveset A, Chamorro S, Olesen JM, Heleno R. Space, time and aliens: charting the dynamic structure of Galápagos pollination networks. AoB Plants. 2015;7: plv068. doi:10.1093/aobpla/plv068

134. Traveset A, Richardson DM. Mutualistic interactions and biological invasions. Annu Rev Ecol Evol Syst. 2014;45: 89–113. doi:10.1146/annurev-ecolsys-120213-091857

135. Bartlett BR. The Influence of Ants Upon Parasites, Predators, and Scale Insects. Ann Entomol Soc Am. 1961;54: 543–551. doi:10.1093/aesa/54.4.543

136. Telfer S, Bown KJ, Sekules R, Begon M, Hayden T, Birtles R. Disruption of a host-parasite system following the introduction of an exotic host species. Parasitology. 2005;130: 661–668. doi:10.1017/S0031182005007250

137. Thieltges DW, Reise K, Prinz K, Jensen KT. Invaders interfere with native parasite-host interactions. Biol Invasions. 2009;11: 1421–1429. doi:10.1007/s10530-008-9350-y

138. Lettoof DC, Greenlees MJ, Stockwell M, Shine R. Do invasive cane toads affect the parasite burdens of native Australian frogs? Int J Parasitol Parasites Wildl. 2013;2: 155–164. doi:10.1016/j.ijppaw.2013.04.002

139. Nelson FBL, Brown GP, Shilton C, Shine R. Helpful invaders: Can cane toads reduce the parasite burdens of native frogs? Int J Parasitol Parasites Wildl. 2015;4: 295–300. doi:10.1016/j.ijppaw.2015.05.004

140. Loxton KC, Lawton C, Stafford P, Holland C V. Parasite dynamics in an invaded ecosystem: Helminth communities of native wood mice are impacted by the invasive bank vole. Parasitology. 2017;144: 1476–1489. doi:10.1017/S0031182017000981

141. Tierney PA, Caffrey JM, Vogel S, Matthews SM, Costantini E, Holland C V. Invasive freshwater fish (*Leuciscus leuciscus*) acts as a sink for a parasite of native brown trout Salmo trutta. Biol Invasions. 2020;22: 2235–2250. doi:10.1007/s10530-020-02253-1

142. Constible JM, Sweitzer RA, Van Vuren DH, Schuyler PT, Knapp DA. Dispersal of non-native plants by introduced bison in an island ecosystem. Biol Invasions. 2005;7: 699–709. doi:10.1007/s10530-004-5859-x

143. Auffret AG. Can seed dispersal by human activity play a useful role for the conservation of European grasslands? Appl Veg Sci. 2011;14: 291–303. doi:10.1111/j.1654-109X.2011.01124.x

144. Bullock JM, Galsworthy SJ, Manzano P, Poschlod P, Eichberg C, Walker K, et al. Process-based functions for seed retention on animals: A test of improved descriptions of dispersal using multiple data sets. Oikos. 2011;120: 1201–1208. doi:10.1111/j.1600-0706.2010.19092.x

145. Cox PA. Extinction of the Hawaiian Avifauna Resulted in a Change of Pollinators for the ieie, *Freycinetia arborea*. Oikos. 1983;41: 195. doi:10.2307/3544263

146. Gross CL. The effect of introduced honeybees on native bee visitation and fruit-set in *Dillwynia juniperina* (Fabaceae) in a fragmented ecosystem. Biol Conserv. 2001;102: 89–95. doi:10.1016/S0006-3207(01)00088-X

147. Dungan RJ, O’Cain MJ, Lopez ML, Norton DA. Contribution by possums to seed rain and subsequent seed germination in successional vegetation, Canterbury, New Zealand. N Z J Ecol. 2002;26: 121–128.

148. Ness JH, Bronstein JL. The effects of invasive ants on prospective ant mutualists. Biol Invasions. 2004;6: 445–461. doi:10.1023/B:BINV.0000041556.88920.dd

149. Abe T, Wada K, Kato Y, Makino S, Okochi I. Alien pollinator promotes invasive mutualism in an insular pollination system. Biol Invasions. 2011;13: 957–967. doi:10.1007/s10530-010-9882-9

150. Young LM. Seed dispersal mutualisms and plant regeneration in New Zealand alpine ecosystems. University of Canterbury. 2012.

151. Hernández-Brito D, Romero-Vidal P, Hiraldo F, Blanco G, Díaz-Luque JA, Barbosa JM, et al. Epizoochory in parrots as an overlooked yet widespread plant–animal mutualism. Plants. 2021;10: 1–11. doi:10.3390/plants10040760

152. Binggeli P, Eakin M, Macfadyen A, Power J, McConnell J. Impact of the alien sea buckthorn (*Hippophae rhamnoides* L.) on sand dune ecosystems in Ireland. Coast dunes Proc 3rd Eur dune Congr Galway, 1992. 1992;1989: 325–337.

153. Sherley GH, Hayes LM. The conservation of a giant weta (*Deinacrida* n. sp. orthoptera: Stenopelmatidae) at mahoenui, king country: Habitat use, and other aspects of its ecology. New Zeal Entomol. 1993;16: 55–68. doi:10.1080/00779962.1993.9722652

154. Sogge MK, Sferra SJ, Paxton EH. Tamarix as Habitat for Birds. Restor Ecol. 2008;16: 146–154.

155. Norkko J, Reed DC, Timmermann K, Norkko A, Gustafsson BG, Bonsdorff E, et al. A welcome can of worms? Hypoxia mitigation by an invasive species. Glob Chang Biol. 2012;18: 422–434. doi:10.1111/j.1365-2486.2011.02513.x

156. Plaza Cazón J, Viera M, Donati E, Guibal E. Zinc and cadmium removal by biosorption on *Undaria pinnatifida* in batch and continuous processes. J Environ Manage. 2013;129: 423–434. doi:10.1016/j.jenvman.2013.07.011

157. Kang YH, Shin JA, Kim MS, Chung IK. A preliminary study of the bioremediation potential of *Codium fragile* applied to seaweed integrated multi-trophic aquaculture (IMTA) during the summer. J Appl Phycol. 2008;20: 183–190. doi:10.1007/s10811-007-9204-5

158. Armitage KB. The evolution, ecology, and systematics of marmots. Oecologia Mont. 2000;9: 1–18. Retrieved from http://om.vuvb.uniza.sk/index.php/OM/article/view/116

159. Barrio IC, Herrero J, Bueno CG, López BC, Aldezabal A, Campos-Arceiz A, et al. The successful introduction of the alpine marmot *Marmota marmota* in the Pyrenees, Iberian Peninsula, Western Europe. Mamm Rev. 2013;43: 142–155. doi:10.1111/j.1365-2907.2012.00212.x

160. Zhu B, Fitzgerald DG, Mayer CM, Rudstam LG, Mills EL. Alteration of ecosystem function by zebra mussels in Oneida Lake: Impacts on submerged macrophytes. Ecosystems. 2006;9: 1017–1028. doi:10.1007/s10021-005-0049-y

161. Potgieter LJ, Wilson JRU, Strasberg D, Richardson DM. *Casuarina* Invasion Alters Primary Succession on Lava Flows on La Réunion Island. Biotropica. 2014;46: 268–275. doi:10.1111/btp.12103

162. Hernández-Brito D, Carrete M, Blanco G, Romero-Vidal P, Senar JC, Mori E, et al. The role of monk parakeets as nest-site facilitators in their native and invaded areas. Biology (Basel). 2021;10. doi:10.3390/biology10070683

163. Prado P, Peñas A, Ibáñez C, Cabanes P, Jornet L, Álvarez N, et al. Prey size and species preferences in the invasive blue crab, *Callinectes sapidus*: Potential effects in marine and freshwater ecosystems. Estuar Coast Shelf Sci. 2020;245. doi:10.1016/j.ecss.2020.106997

164. Lederer A, Massart J, Janssen J. Impact of round gobies (*Neogobius melanostomus*) on dreissenids (*Dreissena polymorpha* and *Dreissena bugensis*) and the associated macroinvertebrate community across an invasion front. J Great Lakes Res. 2006;32: 1–10. doi:10.3394/0380-1330(2006)32[1:IORGNM]2.0.CO;2

165. Michailidis N, Corrales X, Karachle PK, Chartosia N, Katsanevakis S, Sfenthourakis S. Modelling the role of alien species and fisheries in an Eastern Mediterranean insular shelf ecosystem. Ocean Coast Manag. 2019;175: 152–171. doi:10.1016/j.ocecoaman.2019.04.006

166. Stiles JH, Jones RH. Top-down control by the red imported fire ant (*Solenopsis invicta*). Am Midl Nat. 2001;146: 171–185. doi:10.1674/0003-0031(2001)146[0171:TDCBTR]2.0.CO;2

167. Thieltges DW. Benefit from an invader: American slipper limpet *Crepidula fornicata* reduces star fish predation on basibiont European mussels. Hydrobiologia. 2005;541: 241–244. doi:10.1007/s10750-004-4671-z

168. Mondor EB, Addicott JF. Do exaptations facilitate mutualistic associations between invasive and native species? Biol Invasions. 2007;9: 623–628. doi:10.1007/s10530-006-9062-0

169. Veldtman R, Lado TF, Botes A, Procheş Ş, Timm AE, Geertsema H, et al. Creating novel food webs on introduced Australian acacias: Indirect effects of galling biological control agents. Divers Distrib. 2011;17: 958–967. doi:10.1111/j.1472-4642.2011.00781.x

170. Bjerknes AL, Totland Ø, Hegland SJ, Nielsen A. Do alien plant invasions really affect pollination success in native plant species? Biol Conserv. 2007;138: 1–12. doi:10.1016/j.biocon.2007.04.015

171. IUCN. Guidelines for using the IUCN Environmental Impact Classification for Alien Taxa ( EICAT ) Categories and Criteria. 2020;1.

172. Probert AF, Volery L, Kumschick S, Vimercati G, Bacher S. Understanding uncertainty in the Impact Classification for Alien Taxa (ICAT) assessments. NeoBiota. 2020;62: 387–405. doi:10.3897/neobiota.62.52010

173. Strauss SY, Agrawal AA. The ecology and evolution of plant tolerance to herbivory. Trends Ecol Evol. 1999;14: 179–185. doi:10.1016/S0169-5347(98)01576-6

174. Garcia LC, Eubanks MD. Overcompensation for insect herbivory: a review and meta-analysis of the evidence. Ecology. 2019;100: 1–14. doi:10.1002/ecy.2585

175. Clayton D, Moore J. Host-parasite evolution. Oxford, UK: Oxford University Press; 1997. Available: https://agris.fao.org/agris-search/search.do?recordID=US201300024637

176. Agrawal AA. Overcompensation of plants in response to herbivory and the by-product benefits of mutualism. Trends Plant Sci. 2000;5: 309–313. doi:10.1016/S1360-1385(00)01679-4

177. Schröder A, van Leeuwen A, Cameron TC. When less is more: Positive population-level effects of mortality. Trends Ecol Evol. 2014;29: 614–624. doi:10.1016/j.tree.2014.08.006

178. Abrams PA. When does greater mortality increase population size? the long history and diverse mechanisms underlying the hydra effect. Ecol Lett. 2009;12: 462–474. doi:10.1111/j.1461-0248.2009.01282.x

179. Chan WY, Hoffmann AA, van Oppen MJH. Hybridization as a conservation management tool. Conserv Lett. 2019;12: 1–11. doi:10.1111/conl.12652

180. Todesco M, Pascual MA, Owens GL, Ostevik KL, Moyers BT, Hübner S, et al. Hybridization and extinction. Evol Appl. 2016;9: 892–908. doi:10.1111/eva.12367

181. Volery L, Bacher S, Blackburn TM, Bertolino S, Evans T, Genovesi P, et al. Improving the Environmental Impact Classification for Alien Taxa (EICAT): a summary of revisions to the framework and guidelines. NeoBiota. 2020;62: 547–567. doi:10.3897/neobiota.62.52723

182. Stelkens RB, Brockhurst MA, Hurst GDD, Greig D. Hybridization facilitates evolutionary rescue. Evol Appl. 2014;7: 1209–1217. doi:10.1111/eva.12214

183. Frankham R. Genetic rescue of small inbred populations: meta-analysis reveals large and consistent benefits of gene flow. Mol Ecol. 2015;24: 2610–2618. doi:10.1111/mec.13139
